# EGFR amplification and EGFRvIII predict and participate in TAT-Cx43_266–283_ antitumor response in preclinical glioblastoma models

**DOI:** 10.1093/neuonc/noae060

**Published:** 2024-03-20

**Authors:** Andrea Álvarez-Vázquez, Laura San-Segundo, Pilar Cerveró-García, Raquel Flores-Hernández, Claudia Ollauri-Ibáñez, Berta Segura-Collar, Christopher G Hubert, Gillian Morrison, Steven M Pollard, Justin D Lathia, Pilar Sánchez-Gómez, Arantxa Tabernero

**Affiliations:** Department of Biochemistry and Molecular Biology, Neuroscience Institute of Castilla y León (INCyL), Institute for Biomedical Research of Salamanca (IBSAL), University of Salamanca, Salamanca, Spain; Centre for Cancer Research-IBMCC (CSIC), IBSAL, Salamanca, Spain; Department of Biochemistry and Molecular Biology, Neuroscience Institute of Castilla y León (INCyL), Institute for Biomedical Research of Salamanca (IBSAL), University of Salamanca, Salamanca, Spain; Department of Biochemistry and Molecular Biology, Neuroscience Institute of Castilla y León (INCyL), Institute for Biomedical Research of Salamanca (IBSAL), University of Salamanca, Salamanca, Spain; Department of Biochemistry and Molecular Biology, Neuroscience Institute of Castilla y León (INCyL), Institute for Biomedical Research of Salamanca (IBSAL), University of Salamanca, Salamanca, Spain; Instituto de investigaciones Biomédicas I+12 (Imas12), Hospital 12 de Octubre, Madrid, Spain; Department of Biochemistry, Case Western Reserve University, Cleveland, Ohio, USA; Centre for Regenerative Medicine, Institute for Regeneration and Repair, University of Edinburgh, Edinburgh, UK; Centre for Regenerative Medicine, Institute for Regeneration and Repair, University of Edinburgh, Edinburgh, UK; Department of Cardiovascular & Metabolic Sciences, Lerner Research Institute, Cleveland Clinic, Cleveland, Ohio, USA; Cleveland Clinic Lerner College of Medicine, Case Western Reserve University, Cleveland, Ohio, USA; Neuro-Oncology Unit, Instituto de Salud Carlos III (ISCIII-UFIEC), Madrid, Spain; Department of Biochemistry and Molecular Biology, Neuroscience Institute of Castilla y León (INCyL), Institute for Biomedical Research of Salamanca (IBSAL), University of Salamanca, Salamanca, Spain

**Keywords:** cell-penetrating peptides, EGFR, glioblastoma, NSCs, Src

## Abstract

**Background:**

Glioblastoma (GBM) commonly displays epidermal growth factor receptor (EGFR) alterations (mainly amplification and EGFRvIII) and TAT-Cx43_266–283_ is a Src-inhibitory peptide with antitumor properties in preclinical GBM models. Given the link between EGFR and Src, the aim of this study was to explore the role of EGFR in the antitumor effects of TAT-Cx43_266–283_.

**Methods:**

The effect of TAT-Cx43_266–283_, temozolomide (TMZ), and erlotinib (EGFR inhibitor) was studied in patient-derived GBM stem cells (GSCs) and murine neural stem cells (NSCs) with and without EGFR alterations, in vitro and in vivo. EGFR alterations were analyzed by western blot and fluorescence in situ hybridization in these cells, and compared with Src activity and survival in GBM samples from The Cancer Genome Atlas.

**Results:**

The effect of TAT-Cx43_266–283_ correlated with EGFR alterations in a set of patient-derived GSCs and was stronger than that exerted by TMZ and erlotinib. In fact, TAT-Cx43_266-283_ only affected NSCs with EGFR alterations, but not healthy NSCs. EGFR alterations correlated with Src activity and poor survival in GBM patients. Finally, tumors generated from NSCs with EGFR alterations showed a decrease in growth, invasiveness, and vascularization after treatment with TAT-Cx43_266–283,_ which enhanced the survival of immunocompetent mice.

**Conclusions:**

Clinically relevant EGFR alterations are predictors of TAT-Cx43_266–283_ response and part of its mechanism of action, even in TMZ- and erlotinib-resistant GSCs. TAT-Cx43_266–283_ targets NSCs with GBM-driver mutations, including EGFR alterations, in an immunocompetent GBM model in vivo, suggesting a promising effect on GBM recurrence. Together, this study represents an important step toward the clinical application of TAT-Cx43_266–283_.

## Graphical Abstract

**Graphical Abstract:**
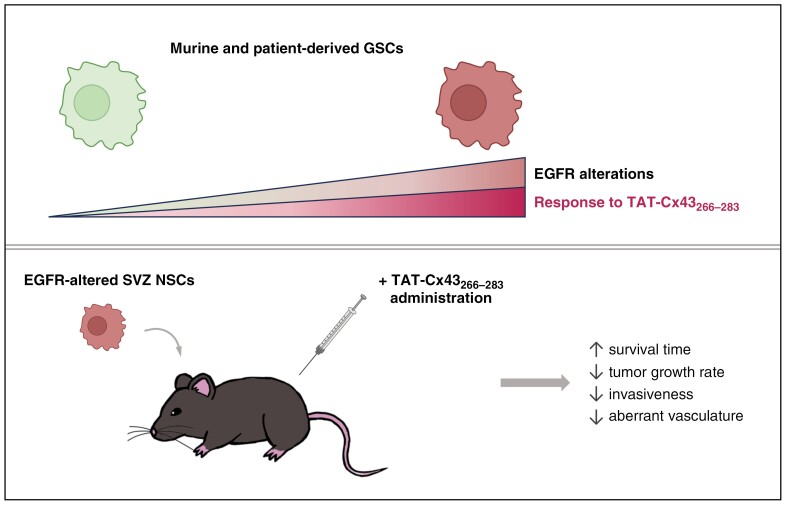

**Key Points**

- Epidermal growth factor receptor (EGFR) and EGFRvIII predict and participate in TAT-Cx43_266–283_ response in glioblastomas (GBMs).
- TAT-Cx43_266–283_ targets temozolomide- and erlotinib-resistant GBM stem cells.
- TAT-Cx43_266–283_ targets neural stem cells with GBM-driver mutations in vitro and in vivo.

**Importance of the Study**

TAT-Cx43_266–283_ is a Src inhibitor with antitumor properties in preclinical glioblastoma (GBM) models. Here, we reveal that epidermal growth factor receptor (EGFR) amplification and EGFRvIII predict TAT-Cx43_266–283_ response in GBMs. The high frequency of these alterations suggests that TAT-Cx43_266–283_ could be beneficial to numerous GBM patients, as shown in our results in patient-derived GBM samples. In addition, the identification of biomarkers of treatment response might help patient stratification for a future clinical trial. This study confirms the clinical relevance of the EGFR-Src axis and its involvement in the antitumor mechanism of action of TAT-Cx43_266–283_, which may explain its effect in temozolomide- and erlotinib-resistant GBM stem cells. Importantly, TAT-Cx43_266–283_ targeted neural stem cells with GBM driver mutations, reduced the rate of tumor growth and GBM hallmarks, enhancing the survival of immunocompetent GBM-bearing mice. These results suggest a potential benefit in GBM recurrence. Together, our results support the clinical application of TAT-Cx43_266–283_ against GBM.

Glioblastoma (GBM) is the most common and aggressive primary malignant brain tumor, with a poor survival.^1^ Despite the advances in basic and clinical research, it remains an incurable cancer with an urgent need for new therapies. The epidermal growth factor (EGF) receptor (EGFR), also named erbB1, which responds to extracellular ligands such as EGF and TGF-α, was one of the first proto-oncogenes identified to play a potential role in GBM pathogenesis because of its high level of expression in the majority of patients and frequent genomic amplification.^2,3^ In fact, according to the 2021 WHO Classification of Tumors of the CNS, the presence of EGFR amplification in isocitrate dehydrogenase (IDH)-wildtype diffuse astrocytomas is sufficient to diagnose GBM.^1^ Among the most common genetic alterations found in GBM, together with the amplification of EGFR, is its truncated version EGFRvIII, which lacks the extracellular domain and is constitutively active.^4^ Analyses of the clonal evolution of the major EGFR aberrations showed that EGFR amplification is an early event and that EGFRvIII mutations emerge from intratumoral heterogeneity later in tumor development.^5^ Moreover, it has been shown that independently of EGFR amplification status, the EGFR pathway is overexpressed in recurrent tumors, making EGFR a key protein also in recurrence,^6^ which occurs in most GBM patients.

GBMs are composed of a heterogeneous population of cells, including some with stem-cell-like properties, known as GBM-initiating cells or GBM stem cells (GSCs). GSCs are characterized by their self-renewal capacity, high oncogenic potential, and resistance to standard therapies, such as tumor resection combined with radiation or temozolomide (TMZ).^7^ Several lines of evidence suggest that some GBMs originate from neural stem cells (NSCs). Thus, it has been reported that mutations in NSCs from the subventricular zone (SVZ) in mice, but not in other neural cells, promote GBM development.^8–11^ In line with this, Lee et al. found that human GBM cells share critical mutations for GBM development with NSCs from the SVZ, suggesting that GBM cells arise from NSCs with driver mutations.^12^ Interestingly, EGFR is one of the proteins whose alteration has been linked to the transition from NSCs to GSCs.^13^ On that note, NSCs with EGFR and/or other GBM driver-mutations are commonly studied as they provide important clues about GBM initiation, progression, and recurrence.^9,11,14,15^

GBMs and, particularly, GSCs, display high oncogenic activity of Src,^16^ which is a non-receptor tyrosine kinase that positively modulates a variety of cellular functions related to cancer, including proliferation, invasion, motility, survival, differentiation, angiogenesis, and metabolic reprogramming.^17,18^ Src is rarely mutated in human GBM, but it is significantly overactivated.^19^ Indeed, GBM shows the highest overactivation of Src across many tumor types.^18^ Not surprisingly, lower activity of Src in GBM patients correlated with better progression-free survival.^20^ Src is activated in response to a great diversity of signals, including those triggered by oncogenic growth factor receptors, such as EGFR and EGFRvIII, which activate Src and its downstream pathways involved in cancer progression.^5,21,22^ Src also activates EGFR^23^ creating a reciprocal activation loop. Thus, Src may phosphorylate EGFR and EGFRvIII triggering their downstream signaling^5,24–27^ associated with oncogenesis and tumor progression.^28,29^ Therefore, the interaction between Src and EGFR/EGFRvIII is involved in cell transformation, and it is considered an interesting target for cancer therapy.^5,28,30,31^

Connexin43 (Cx43), the gap junction forming protein, physiologically inhibits Src activity^32^ through the recruitment of Src and its endogenous inhibitors, CSK and PTEN.^33^ The Cx43-mimetic peptide, TAT-Cx43_266–283_, recapitulates this mechanism because it acts as a docking platform for Src, CSK, and PTEN. Consequently, TAT-Cx43_266–283_ is a specific inhibitor of Src^34^ with important antitumor effects in several preclinical models of GBM in vitro, ex vivo, and in vivo, including freshly removed surgical specimens from patients.^34,35^ Tumor cell proliferation, survival, migration, invasion, metabolic plasticity, and autophagy are impaired by TAT-Cx43_266–283_, with the subsequent improvement of survival of GBM-bearing mice.^20,35–37^ One of the main advantages of TAT-Cx43_266–283_ is that it specifically targets GSCs, and has no toxic effects in healthy brain cells.^37^ Indeed, the toxicity of TAT-Cx43_266–283_ in neurons and astrocytes is much lower than that exerted by Dasatinib.^36^

Due to the high frequency of EGFR alterations found in human GBMs and the participation of Src activity in EGFR signaling, we explored a possible link of EGFR alterations with the effect of the Src inhibitor TAT-Cx43_266-283_ in GSCs and NSCs with or without EGFR alterations.

## Methods

For detailed protocols, see Supplementary information.

### Animals

Equal number of male and female C57BL/6 mice were obtained from Charles River Laboratories and the animal facility of the University of Salamanca. Animal procedures were approved by the ethics committee of the University of Salamanca and the Junta de Castilla y León (Spain) and were carried out in accordance with European Community Council directives (2010/63/UE), and Spanish law (RD 53/2013 BOE 34/11370–420, 2013).

### Cell Culture

SVZ-NSCs, SVZ-EGFRwt, and SVZ-EGFRvIII cells were generated by the Neuro-Oncology Unit (Instituto de Salud Carlos III, Madrid, Spain) by retroviral expression of EGFRwt or EGFRvIII in primary SVZ NSCs from p16/p19 K.O. mice and were cultured as described.^15^

NSC-EGFRvIII, NP, NPE, and NPE-IE cells, and the patient-derived GSCs E15, E20, E22, E26, E28, E43, and E51 were obtained from the Glioma Cellular Genetics Resource (University of Edinburgh, Edinburgh). NSC-EGFRvIII, NP, NPE, and NPE-IE cells were obtained by genetically modifying SVZ NSCs from C57BL/6 mice.^9^ NP, NPE, and NPE-IE cell lines express GFP and luciferase. Patient-derived GSCs L0, L1, and L2 were obtained from the University of Florida, T4121 cells from Duke University, DI318 cells from Cleveland Clinic, 23M cells from University of Texas/MD Anderson Cancer Center and control immortalized hNSC from Fred Hutchinson Cancer Center/University of Washington. These cells were cultured in GSC medium in adherent conditions.

### Treatments

Peptides (>95% pure; GenScript) were used as described.^20,35–37^ TAT-Cx43_266–283_ and TAT-Cx43_274–291_ sequences were TAT-AYFNGCSSPTAPLSPMSP (patent ID: ES2526109B1)^34^ and TAT-PTAPLSPMSPPGYKLVTG, respectively. TAT (YGRKKRRQRRR) and TAT-Cx43_274–291_ were used as control peptides, as they do not inhibit Src activity or exert antitumor effects.^20,33,35^

For in vitro experiments, peptides were used at 50 μM in the culture medium. Unless otherwise stated, temozolomide was used at 100 μM and erlotinib at 1 µM in the culture medium.

For in vivo experiments, 100 μM TAT-Cx43_266–283_ or GSC medium were injected intracranially with tumor cells. One week after the injection, either 4 nmol/g TAT-Cx43_266–283_ or 0.9% NaCl were intraperitoneally injected twice a week for the duration of the experiment as reported.^36^

### Alamar Blue Viability Assay

Either 2500 or 5000 cells per well (96-well plates) were plated, treated, and 10% Alamar blue/resazurin was added at the indicated times.

### Intracranial Injection of GSCs

NPE-IE cells (200 000) were intracranially injected in the right striatum (0.5 mm AP, +1.5 LM to bregma, and −2.5 mm deep) of 8-week-old C57BL/6 mice as described.^9^

Bioluminescence imaging was performed approximately every 2 weeks using an IVIS Lumina S5 (Perkin Elmer). The highest signal in photons/s (p/s) was selected as the measure of luciferase activity. Values > 8 × 10^8^ p/s were used as an endpoint criterion.

Animals that did not show luciferase activity at day 27 post injection, and signs of tumor presence (humane endpoint signs) were not included in the study. On that note, <50% of injected mice developed tumors, despite following the protocol previously described.^9^

### Immunofluorescence

Brain sections were incubated with a rat anti-CD31 monoclonal antibody (1:100, BD Biosciences, ref: 550274) or a mouse anti-VEGF monoclonal antibody (1:50, Santa Cruz Biotechnology, ref: sc-7269). Mosaic images were acquired, using a Leica Stellaris 8 confocal microscope, processed, and analyzed using LAS X (Leica) and Fiji.

### Blood Vessel Analyses

Mean lacunarity and vessel area (CD31 area) were analyzed in CD31 confocal mosaic images using AngioTool 0.6a (N.C.I., USA). Blood vessels were selected, non-specific small particles were removed and mean lacunarity was measured. Mean lacunarity × vessel area was used to correct differences in the number of vessels per region.

The number and area of large blood vessels (areas ≥ 0.1 mm^2^) were quantified in CD31 confocal mosaic images using Fiji.

### Migration and Invasion Assays

Migration and invasion assays in vitro were carried out as described.^35^ For invasion experiments, 75 000 cells with treatments, were plated in the upper chamber of Matrigel-coated inserts and DMEM/F12 + 10% FBS was added to the lower well. After 15 h, the invading cells, stained with Giemsa, were counted.

For migration experiments, 5000 cells/well (24-well plates) were recorded using the time-lapse live cell imaging microscope Zeiss Axio Observer Z1 coupled to an AxioCam MRm camera, which acquired phase-contrast photographs every 10 min for 24 h. Mean velocity was obtained using Fiji.

The invasiveness of GFP^+^ NPE-IE cells in vivo was determined by thresholding GFP fluorescence mosaic images, establishing the tumor rims, and quantifying the area occupied by GFP^+^ NPE-IE cells infiltrating the brain parenchyma using Fiji.

### Western Blots (WB)

Primary antibodies used for WB were: anti-phospho-EGFR Y1068 (1:1000, Cell Signaling, ref: 3777) and anti-EGFR (1:1000, Cell Signaling, ref: 4267), anti-VEGF (1:200, Santa Cruz Biotechnology, ref: sc-7269), anti-phospho-Src Y416 (1:250, Cell Signaling, ref: 2101), anti-Src (1:500, Cell Signaling, ref: 2110), anti-GAPDH (1:5000, Invitrogen, ref: AM4300), and anti-β-actin (1:1000, Sigma, ref: A5441).

### Patient Protein Levels, RNA Expression, and Survival Analysis

The Cancer Genome Atlas (TCGA) GBM database^38^ (392 patients with IDH wildtype GBM) containing reverse phase protein array (RPPA) data (162 patients) was used. cBioportal^39^ was used for downloading data, which was plotted and analyzed using GraphPad Prism 9.0.1.

### Statistical Analysis

Results are expressed as the means ± S.E.M of at least three independent experiments and were plotted in bars with individual data points. Two-tailed Student’s *t*-test and one- or 2-way ANOVA, followed by a post hoc Tukey test were used for comparisons between 2 groups or more than 2 groups, respectively. For survival analyses, data were represented in a Kaplan–Meier curve and differences were compared using a log-rank test.

## Results

### The Effect of TAT-Cx43_266-283_ in GSCs Correlates With Their EGFR Status

**Figure 1.**
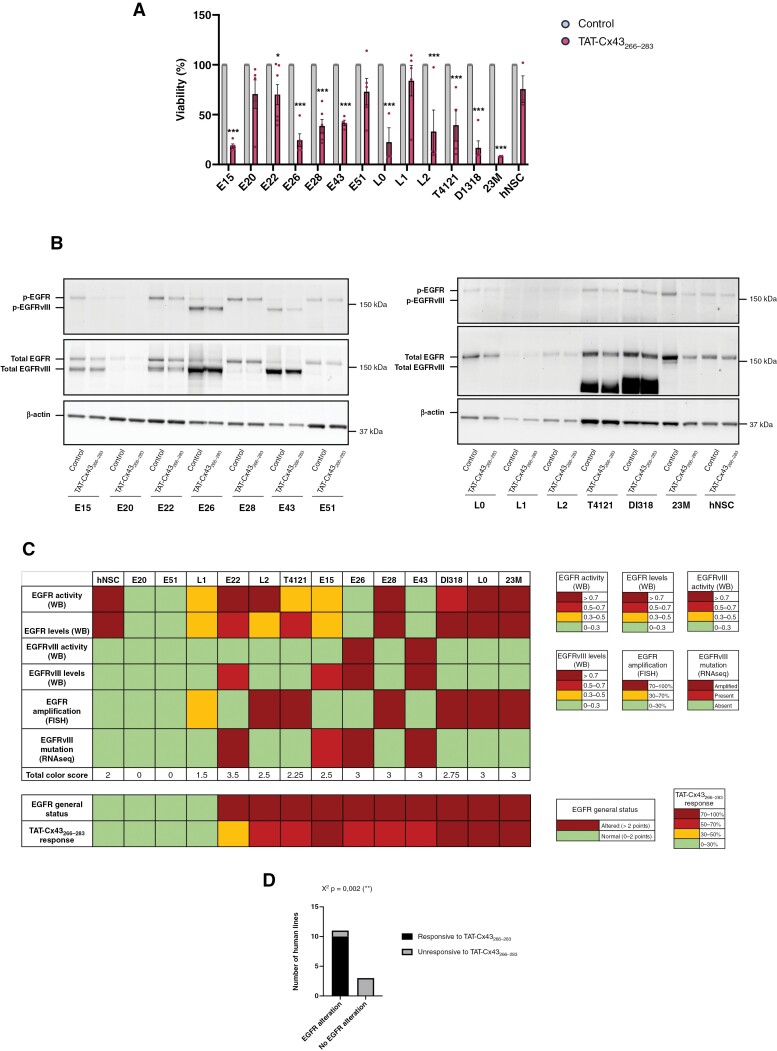
Correlation of EGFR status vs TAT-Cx43_266–283_ response in patient-derived GSCs. A set of GBM patient-derived GSCs and healthy human NSCs were treated with 50 µM TAT-Cx43_266–283_. (A) Alamar blue viability assay after 6 days of treatment administered at days 0 and 3. Results are expressed as the percentage of the control (mean ± S.E.M. of at least 3 independent experiments, ANOVA: *P value < .05, ***P value < .001). (B) WB analysis of EGFR and EGFRvIII levels and activity (p-EGFR Y1068 and p-EGFRvIII Y1068) after 24 h treatment. β-actin was used as a loading control. (C) Heatmap summarizing TAT-Cx43_266–283_ response and EGFR alterations of human patient-derived GSCs and hNSCs determined by WB, RNAseq, and FISH. For total color score, every square was assigned a numeric value according to its color. Dark red = 1 point, light red = 0.75 points, yellow = 0.5 points, and green = 0 points. (D) Contingency table graph showing the magnitude of TAT-Cx43_266–283_ response and EGFR status in patient-derived GSCs and healthy human GSCs. *χ*^2^ test: **P value < .01.

Our previous studies showed that TAT-Cx43_266–283_ specifically reduced cell viability in different human and murine GSCs (GliNS2, G166, G179, GL261, and C6) without exerting toxic effects in neurons and astrocytes.^34,35,37^ To test the extent of TAT-Cx43_266–283_ effect in human GBMs, in this study we increased the number of GBM patient-derived GSC lines analyzed. We found that TAT-Cx43_266–283_ significantly reduced cell viability in 10 different GSC lines out of the 13 analyzed (77%; Figure 1A). The absence of toxic effects in human NSCs (hNSC) confirmed the selectivity of TAT-Cx43_266–283_ in tumor cells.

As expected, WB analyses showed that some cell lines exhibited EGFR alterations, either increased levels or increased activity of EGFR or EGFRvIII (Figure 1B, Supplementary Figures S1 and S2A). Thus, several cell lines exhibited increased expression and activity of EGFRvIII (E15, E22, E26, and E43) or other EGFR mutations (T4121, Dl318). EGFRwt overactivation and overexpression were observed in 23M and L0 cells, respectively, when compared to hNSCs. In addition, TAT-Cx43_266–283_ treatment for 24 h significantly reduced the activity of EGFR and EGFRvIII, as well as the amount of these proteins in some of the analyzed GSC lines (Figure 1B, Supplementary Figures S1 and S2B). In fact, this reduction was observed in most of the GSC lines (Figure 1B), although the quantification did not result in significant differences in some of them (Supplementary Figure S2B).

Although EGFR and EGFRvIII protein levels and activity are the effectors for the oncogenic signaling, WB is generally not used in a clinical setting. Instead, FISH or RNAseq are commonly performed. Therefore, GSCs were scored according to their EGFR alterations (Figure 1C), considering their EGFR and EGFRvIII protein levels and activity analyzed by WB (see Figure 1B and Supplementary Figure S1 for WB images and S2A for quantifications), as well as their *EGFR* amplification and EGFRvIII mutations assessed by FISH and RNAseq, respectively (Supplementary Figures S3A and B and S4). In addition, IDH status, MGMT methylation status, together with information about the recurrence of the tumor of origin are provided in Supplementary Figure S3D. It is important to note that while we focused on EGFRvIII when analyzing EGFR-RNAseq data (Supplementary Figure S4), we found that 2 of the patient-derived GSCs (T4121 and DI318) that did not have EGFRvIII, showed an increase in the expression of exons 15–28 of EGFR, as well as in the junctions between them, indicating high levels of alternative splicing forms of EGFR that were confirmed by WB (Figure 1B and Supplementary Figure S1). Different colors were assigned to each EGFR alteration according to the legend shown in Figure 1C. Then, the total color score was calculated for each cell line by adding colored squares (1, 0.75, 0.5, and 0 points were assigned to dark red, light red, yellow, and green squares, respectively). The EGFR general status was considered altered when this score was >2, considering human NSCs as a baseline. TAT-Cx43_266–283_ response was also scored in 4 levels and compared with EGFR general status. The results showed a clear correlation between EGFR general status and TAT-Cx43_266–283_ effect (Figure 1C). Importantly, either EGFR amplification or EGFRvIII mutation correlated with TAT-Cx43_266–283_ response, suggesting that this information may be sufficient to predict TAT-Cx43_266–283_ response. One exception is E22 GSC line, which was characterized by RNAseq as a cell line with EGFRvIII amplification, but unlike similarly characterized cell lines (E26 and E43), WB showed lower EGFRvIII levels and activity in E22 than in E26 and E43 GSCs, which may explain the lower response to TAT-Cx43_266–283_ found in E22. It should be mentioned that the levels and activity of EGFRvIII in E22 GSCs may be downregulated during the passages, as shown in Supplementary Figure S1 where E22 GSCs at passages 5 and 15 were compared, revealing a reduction in EGFRvIII levels throughout the time in culture. Overall, EGFR general status clearly correlated with TAT-Cx43_266–283_ response (Figure 1C). Indeed, the contingency table graph (Figure 1D) and correlation graph (Supplementary Figure S3C) showed a significant positive correlation between EGFR alterations and TAT-Cx43_266–283_ response.

**Figure 2.**
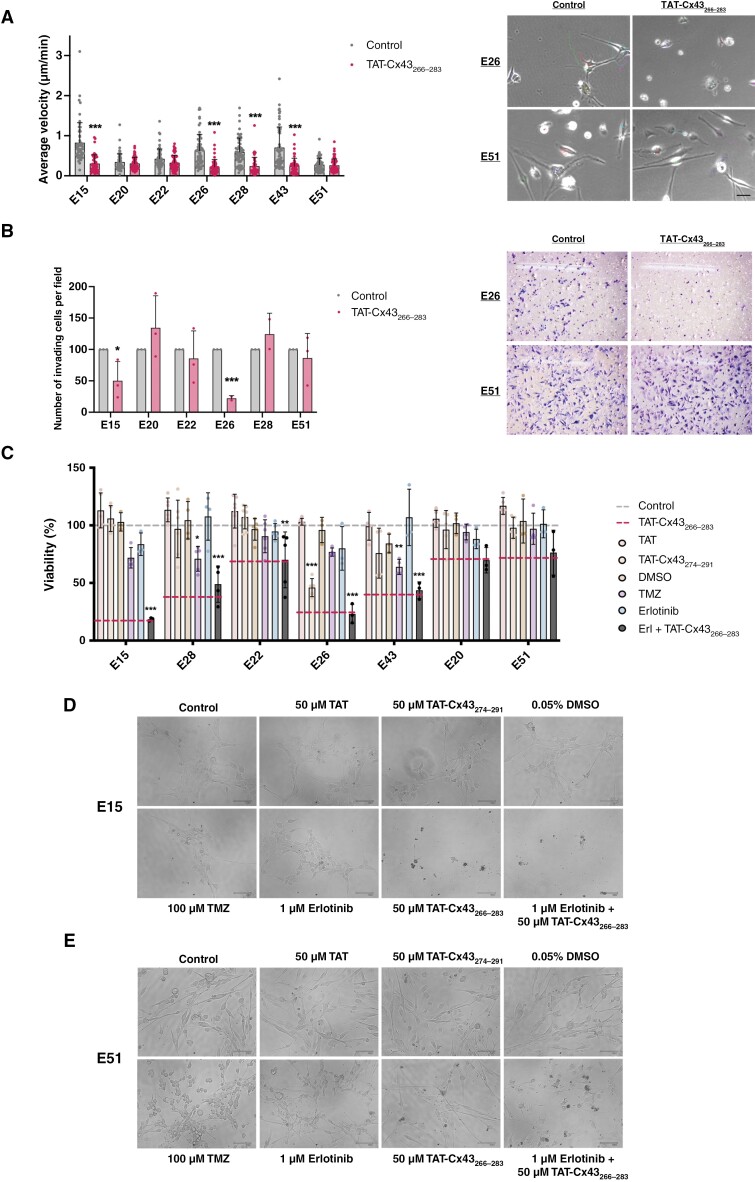
Effect of TAT-Cx43_266–283_ in cell migration and invasion, and comparison of its effect on cell viability vs temozolomide and erlotinib in GBM patient-derived GSCs. A set of GBM patient-derived GSCs and healthy human NSCs were treated with 50 µM TAT, 50 µM TAT-Cx43_274–291_ (2 cell-penetrating peptides used as additional controls), 50 µM TAT-Cx43_266–283_, 100 µM temozolomide, 1 µM erlotinib, 0.05% DMSO (v/v) (vehicle for erlotinib), and the combination of 1 µM erlotinib plus 50 µM TAT-Cx43_266–283_. (A) Quantification of the average velocity of EGFR altered and unaltered patient-derived GSCs after treatment with 50 µM TAT-Cx43_266–283_ and representative frames from the time-lapse microscopy movies of EGFR altered (E26) and EGFR unaltered (E51) patient-derived GSCs (see SupplementaryMovies). Results are represented as mean ± SEM of 2 independent experiments in which approximately 50 cells per cell line and experimental group were tracked, ANOVA: ***P value < .001 vs control. Scale bar: 50 µm. (B) Quantification of the number of Matrigel invading GSCs per frame after treatment with 50 µM TAT-Cx43_266–283_ and representative frames of EGFR altered (E26) and EGFR unaltered (E51) patient-derived GSCs. Results are expressed as the percentage of the control (mean ± SEM of 3 independent experiments except for the lines E26 and E28, in which 2 independent experiments were performed, ANOVA: *P value < .05, ***P value < .001 vs control. (C) Alamar blue viability assay after 6 days of treatment administered at days 0 and 3. Results are expressed as the percentage of the control (upper dashed line) (mean ± SEM of at least 3 independent experiments, ANOVA: *P value < .05, **P value < .01, ***P value < .001 vs control. (D and E) Representative phase-contrast images of 2 patient-derived GSCs with (E15) (D) and without (E51) (E) EGFR alterations treated as described above. Scale bar: 100 µm.

As EGFR alterations are a major driver of GBM invasion^40^ and our previous study showed that TAT-Cx43_266–283_ reduced migration and invasion in human GSCs,^35^ we analyzed migration and invasion in human GSCs with and without alterations in EGFR (Figure 2A and B). Our results show that TAT-Cx43_266–283_ significantly reduced the rate of cell motility in human GSCs with EGFR alterations (E15, E26, E28, and E43; except for E22), without affecting that of EGFR unaltered GSCs (E20 and E51) (Figure 2A and Supplementary Movies S1–S14). When their invasion ability was analyzed using Matrigel transwell system, we found more variable results, although TAT-Cx43_266–283_ significantly reduced the invasion of EGFR-altered cells (E15 and E26) without affecting that of EGFR unaltered GSCs (E20 and E51) (Figure 2B). Together, our results indicate that the effect of TAT-Cx43_266–283_ on GSC viability and migration correlates with EGFR alterations in GSCs.

### The Effect of TAT-Cx43_266-283_ on GBM Patient-Derived GSC Viability is Higher Than That Exerted by Erlotinib or TMZ

Because our data indicate that the Src inhibitor, TAT-Cx43_266–283_, also targeted EGFR in GSCs, we compared the effect of TAT-Cx43_266–283_ with that promoted by erlotinib, a classical EGFR inhibitor, and with TMZ, the standard treatment for GBMs. TAT and TAT-Cx43_274–291_, two cell-penetrating peptides that did not inhibit Src activity or exert antitumor effects in previous studies,^20,33,35^ were used as control peptides and DMSO (erlotinib vehicle) was included as a control for erlotinib (Figure 2C–E). Similarly to what is found in a clinical setting, every patient-derived GSC line showed a different and specific pattern of response to treatments. Importantly, the highest inhibition of cell viability was found in TAT-Cx43_266–283_-treated GBM patient-derived GSC lines (red dashed line), when compared to treatment with erlotinib and TMZ. Erlotinib reduced cell viability in a dose-dependent fashion (Supplementary Figure S6A), however, even at the highest dose tested the effect of erlotinib was lower than that of TAT-Cx43_266–283_ in most GSCs (Supplementary Figure S6B). In addition, some EGFR-altered GSCs (E28 and E43) did not respond to high doses of erlotinib, although they responded to TAT-Cx43_266–283_ (Supplementary Figure S6A). TMZ significantly inhibited the cell viability of two cell lines (E28 and E43) but to a lesser extent than TAT-Cx43_266–283_. In addition, the combination of erlotinib plus TAT-Cx43_266–283_ did not enhance the effect of TAT-Cx43_266–283_. Those cells with no significant changes in cell viability after TAT-Cx43_266–283_ treatment (E20 and E51) were also unaffected by erlotinib or TMZ (Figure 2C and Supplementary Figure S6A).

Figure 2D and E show representative images of E15 (responsive to TAT-Cx43_266–283_) and E51 (unresponsive to TAT-Cx43_266–283_) patient-derived GSC morphology after different treatments. It should be noted that although no significant effects on cell viability were found in E51 GSCs after TAT-Cx43_266–283_ treatment, their morphology seemed to be affected. Supplementary Figure S5 includes representative images of different GSCs after different treatments as well as cell viability at different time points, confirming that TAT-Cx43_266–283_ effect is higher than that exerted by erlotinib and TMZ in human GSCs.

### The Effect of TAT-Cx43_266-283_ in NSCs Depends on EGFR Alterations

As EGFR signaling is involved in the transition from NSCs to GSCs^13^ and TAT-Cx43_266–283_ effects on GSC correlated with EGFR alterations, we investigated the effect of TAT-Cx43_266–283_ on NSCs with different alterations in EGFR.

**Figure 3.**
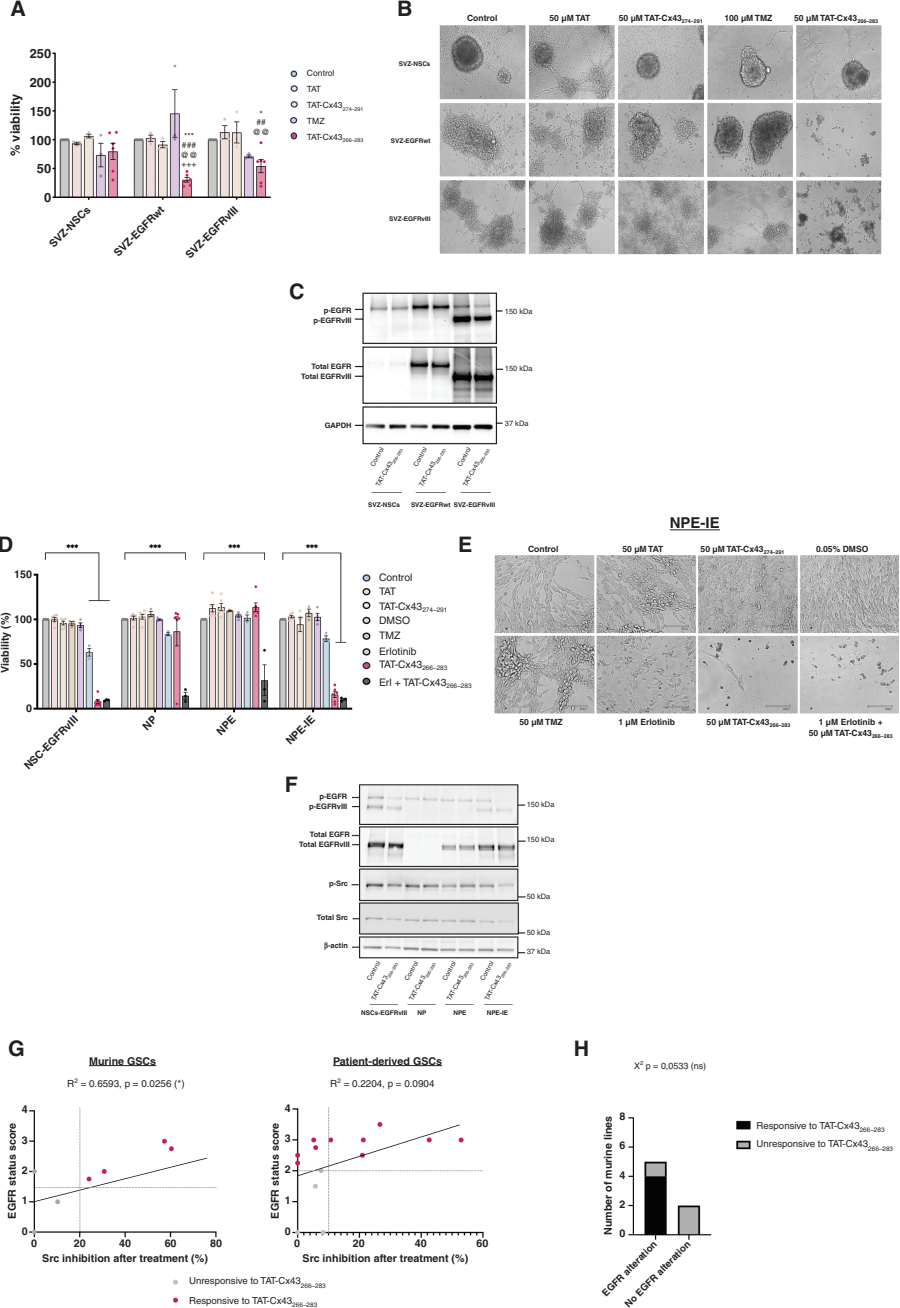
The effect of TAT-Cx43_266–283_ depends on EGFR alterations. A set of murine SVZ NSCs with EGFR alterations (SVZ-EGFRwt and SVZ-EGFRvIII) and a set of murine SVZ NSCs with EGFRvIII mutation (NSC-EGFRvIII), with Nf1/PTEN deletion (NP) or with a combination of Nf1/PTEN deletion and EGFRvIII mutation (NPE, and their immune evasive version, NPE-IE) and their non-modified counterparts (SVZ-NSCs) were treated with 50 µM TAT, 50 µM TAT-Cx43_274–291_ (2 cell-penetrating peptides used as additional controls), 50 µM TAT-Cx43_266–283_, 100 µM temozolomide, 1 µM erlotinib, 0.05% DMSO (v/v) (vehicle for erlotinib) and the combination of 1 µM erlotinib plus 50 µM TAT-Cx43_266–283_. (A) Alamar blue viability assay of SVZ NSCs with EGFR alterations and healthy NSCs after 6 days of treatment administered at days 0 and 3. Results are expressed as the percentage of the control (mean ± SEM of at least 3 independent experiments, ANOVA: *P < .05, ***P < .001 vs control; ##*P* < .01, ###*P* < .001 vs TAT; @@*P* < .01 vs TAT-Cx43_274-291_; +++ *P* < .001 vs temozolomide). (B) Phase-contrast images of murine NSCs with and without EGFR alterations treated as described previously. Scale bar: 100 µm. (C) WB analysis of EGFR and EGFRvIII levels and activity (p-EGFR Y1068 and p-EGFRvIII Y1068) after 24 h treatment. GAPDH was used as a loading control. (D) Alamar blue viability assay of SVZ NSCs with EGFR and additional GBM-driver alterations after 6 days of treatment administered at days 0 and 3. Results are expressed as the percentage of the control (mean ± SEM of at least 3 independent experiments, ANOVA: ***P value < .001). (E) Phase-contrast images of murine SVZ NSCs with GBM-driver alterations treated as described in (A). Scale bar: 100 µm. (F) WB analysis of EGFR, EGFRvIII, and Src levels and activity (p-EGFR Y1068, p-EGFRvIII Y1068, and p-Src Y416) after 24 h treatment. β-actin was used as a loading control. (G) Correlation graph depicting the relationship between GSC EGFR status and Src inhibition after treatment with TAT-Cx43_266–283_ in murine and patient-derived GSCs. (H) Contingency table graph showing the magnitude of TAT-Cx43_266–283_ response and EGFR status of all the murine NSCs and GSCs. *χ*^2^ test was used for statistical analysis.

First, we analyzed TAT-Cx43_266–283_ effect on the cell viability of SVZ NSCs from p16/p19 KO mice (SVZ-NSCs) with overexpression of EGFRwt (SVZ-EGFRwt) or EGFRvIII (SVZ-EGFRvIII).^15^ While 100 µM TMZ did not affect cell viability in SVZ-NSCs with or without EGFR or EGFRvIII, TAT-Cx43_266–283_ only decreased cell viability in NSCs overexpressing EGFR or EGFRvIII, but not in SVZ-NSCs (Figure 3A and B), supporting the role of EGFR signaling in TAT-Cx43_266–283_ effect and confirming the selectivity of TAT-Cx43_266–283_ in tumor cells. It should be mentioned that higher doses of TMZ reduced SVZ-EGFRvIII viability but also that of control SVZ-NSC (Supplementary Figure S11C and D). WBs suggested that TAT-Cx43_266–283_ reduced EGFR and EGFRvIII protein levels and activity in SVZ-EGFRwt and SVZ-EGFRvIII (Figure 3C and Supplementary Figure S7A and B). However, the quantification of these blots showed that only the reduction in the activity of EGFRvIII in SVZ-EGFRvIII was statistically significant (Supplementary Figure S7C).

SVZ-NSCs, SVZ-EGFRwt, and SVZ-EGFRvIII constitute an excellent tool to address the participation of EGFR and EGFRvIII in vitro or in immunocompromised mice.^15^ Therefore, to confirm our results in an immunocompetent murine model of GBM, we studied the effect of TAT-Cx43_266–283_ in a set of GSCs derived from mouse SVZ NSCs engineered with GBM driver mutations, which maintain the ability to generate tumors that recapitulate hallmarks of human GBM in mice.^9^ Thus, we analyzed NSCs with EGFRvIII overexpression (NSCs-EGFRvIII), CRISPR/Cas-mediated ablation of Nf1 and PTEN (NP), CRISPR/Cas-mediated ablation of Nf1 and PTEN and EGFRvIII overexpression (NPE) and NPE-IE cells, which are NPE cells that after serial intracranial transplantations in immunocompetent mice developed immune evasive properties, therefore NPE-IE cells lack Nf1 and PTEN, overexpress EGFRvIII and have immune evasive properties^9^ (Figure 3D–F).

TMZ did not affect the cell viability of any of these cells while erlotinib reduced the viability in NSCs-EGFRvIII, although to a lesser extent than TAT-Cx43_266–283_. Interestingly, the combination of TAT-Cx43_266–283_ and erlotinib strongly reduced NP and NPE cell viability, although neither TAT-Cx43_266–283_ nor erlotinib were effective alone (Figure 3D and E). TAT-Cx43_266–283_ decreased the viability of NSCs-EGFRvIII and NPE-IE cells, while it did not affect that of NP cells (Figure 3D and E and Supplementary Figure S9A and B). These data support the involvement of EGFR in TAT-Cx43_266–283_ effect. Surprisingly, TAT-Cx43_266–283_ did not affect cell viability in NPE cells (Figure 3D and E and Supplementary Figure S9C), even though they were also modified to express EGFRvIII. WB analyses unveiled that NPE cells expressed lower levels of EGFRvIII than NPE-IE and NSCs-EGFRvIII and more importantly, the active form of EGFRvIII was nearly absent in NPE cells (Figure 3F, Supplementary Figures S9D and S10A), which might explain the lack of effect of TAT-Cx43_266–283_ in this cell line. In addition, TAT-Cx43_266–283_ significantly decreased EGFR activity in TAT-Cx43_266–283_-responsive cells, i.e. NSCs-EGFRvIII and NPE-IE cells, while it did not affect EGFR activity in TAT-Cx43_266–283_-unresponsive cells, i.e. NP and NPE cells (Figure 3F and Supplementary Figure S10B).

Interestingly, when we analyzed Src activity, the main target of TAT-Cx43_266–283_, we found that TAT-Cx43_266–283_ only reduced Src activity in TAT-Cx43_266–283_-responsive cells, that is, NSCs-EGFRvIII and NPE-IE (Figure 3F and Supplementary Figure S11A). Src activity was also analyzed in the rest of murine (Supplementary Figure S8) and human cells (Supplementary Figure S12) included in this study. We found a significant correlation between the reduction in Src activity promoted by TAT-Cx43_266–283_ and EGFR alterations in murine cells (Figure 3G and Supplementary Figure S11B). The same trend was observed in patient-derived GSCs (Figure 3G), although it was not statistically significant, probably due to their numerous and heterogeneous molecular alterations compared to specifically engineered murine lines. The lowest Src inhibition (0–10%) was found in TAT-Cx43_266–283_-unresponsive cell lines tested. These results, together with the contingency table graphs (Figures 1D and 3H), support the involvement of the EGFR-Src axis in TAT-Cx43_266–283_ effect.

### EGFR Alterations Correlate With Src Activity in GBM Patients

**Figure 4.**
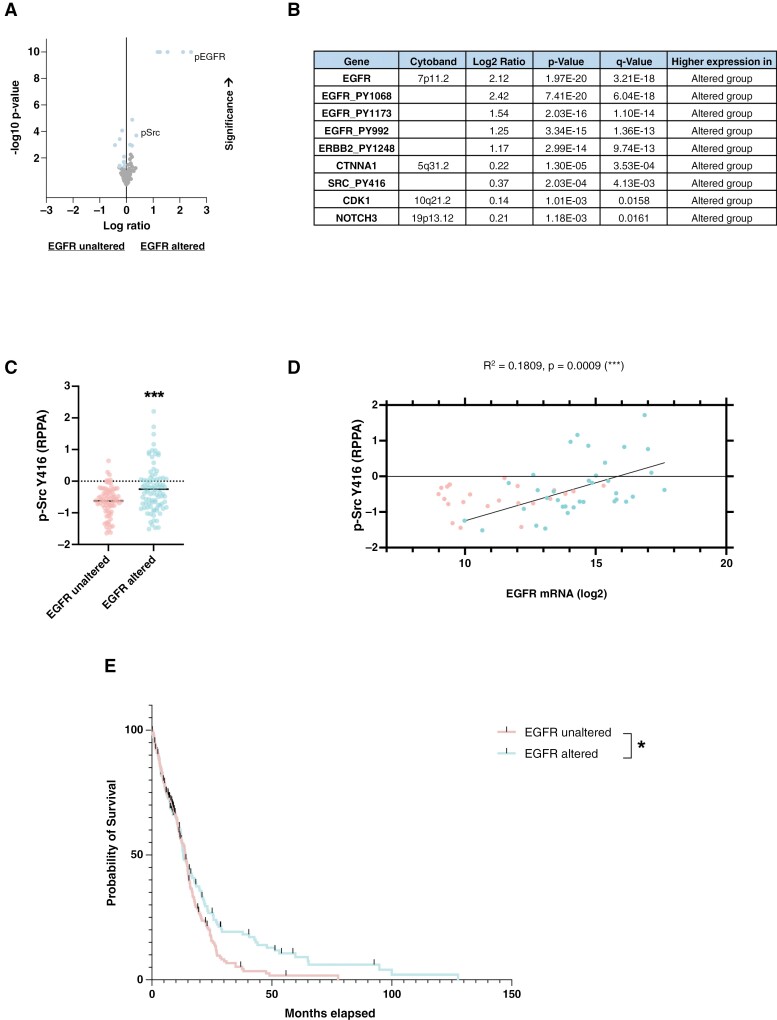
EGFR alterations correlate with Src activity in GBM patients. Analyses were carried out using IDHwt GBM samples from the TCGA GBM database. (A) Volcano plot depicting levels of proteins in GBM patients with or without EGFR alterations. (B) Proteins with significantly higher levels in patients with EGFR alterations (see Supplementary Table S1 for a complete list). (C) Comparative graph of p-Src Y416 levels in patients with and without EGFR alterations (Student’s *t*-test, ***P value < .001). (D) Correlation graph showing the relationship between EGFR mRNA expression and p-Src Y416 protein levels in GBM patients. (E) Overall survival curve of patients with and without EGFR alterations (log-rank test, *P value < .05).

To explore the correlation between EGFR alterations and Src activity and its clinical relevance in GBM patients, we analyzed the dataset of GBM samples provided by The Cancer Genome Atlas (TCGA), which includes genomic, epigenomic, transcriptomic, and proteomic information.^38^ Using cBioportal,^39^ we first filtered IDHwt samples and found that 205 out of 392 samples showed EGFR alterations, including mutations, copy number alterations, and changes in mRNA expression (compared to the expression of tumors that are diploid for *EGFR*, with a *z*-score threshold of ±2) or protein expression (measured by reverse-phase protein array, with a *z*-score threshold of ±2). The comparison of protein levels among these EGFR-altered and unaltered GBM groups showed that the active form of Src (SRC_PY416) is among the top 10 proteins whose abundance is significantly increased in EGFR-altered GBM samples (Figure 4A and B and Supplementary Table S1). Only EGFR, phosphorylated EGFRs and ERBB2 (all of them belonging to Erbb family of receptors), as well as catenin-alpha 1 (CTNNA1) were more abundant than SRC_PY416 in EGFR-altered GBM samples compared to unaltered ones, which highlights the link between EGFR and Src signaling pathways in GBM patients. Indeed, the analysis of these data showed a positive and significant correlation between EGFR alterations and mRNA levels and Src activity (SRC_PY416) in TCGA samples from GBM patients (Figure 4C and D).

To confirm the clinical relevance of these findings, we compared overall survival in these two groups of GBMs. This analysis confirmed that the EGFR alterations correlated with reduced overall survival in GBMs (Figure 4E), which informs about the interest in targeting EGFR alterations linked to higher Src activity in GBMs.

### Effect of TAT-Cx43_266-283_ in an In Vivo GBM Model Derived From NSCs With Alterations in EGFR and Other Glioma-Driver Mutations

**Figure 5.**
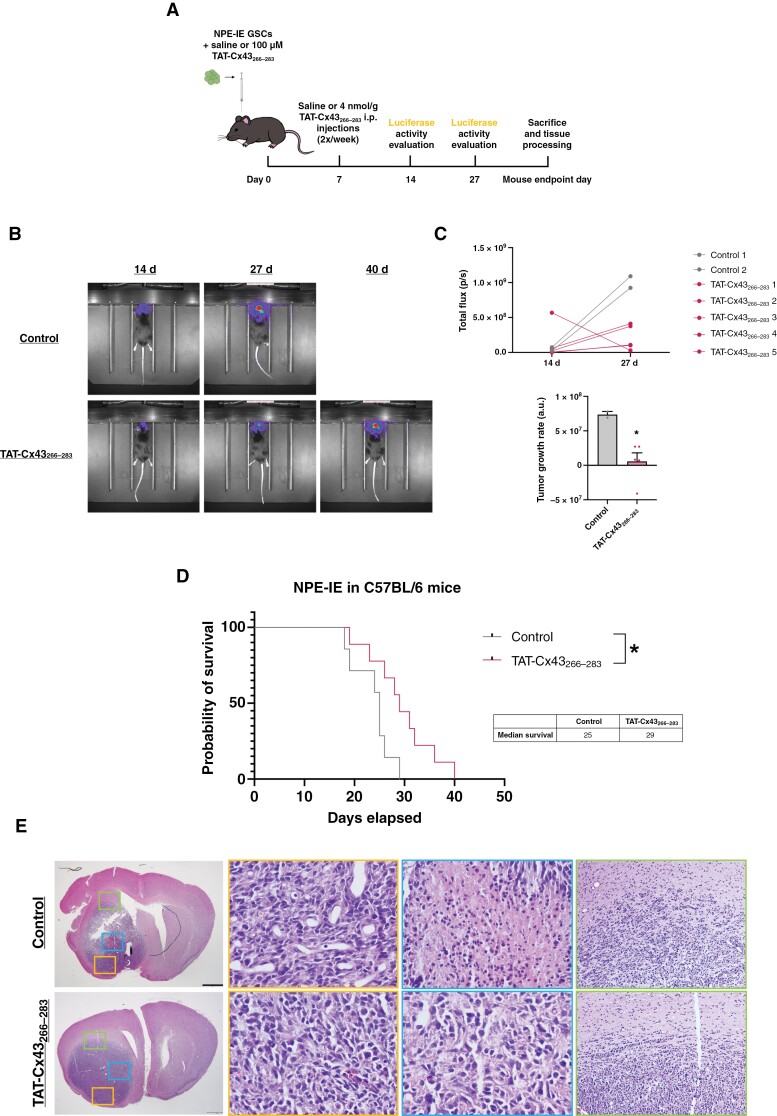
TAT-Cx43_266–283_ enhances the survival of immunocompetent mice bearing GBMs derived from NSCs with alterations in EGFR and other glioma-driver alterations (NPE-IE). GFP-labeled NPE-IE cells together with 100 µM TAT-Cx43_266–283_ or GSC medium were intracranially injected in C57BL/6 mice. After 7 days, a twice per week i.p. injection of saline or 4 nmol/g TAT-Cx43_266–283_ was administered until endpoint signs appeared. (A) Summarizing scheme of the in vivo experiment. (B) In vivo bioluminescence images of control and TAT-Cx43_266–283_-treated tumors 14 days and 27 days after GSC injection. (C) In vivo luciferase activity quantification of individual NPE-IE tumor-bearing animals that lived for at least 27 days, measured in photons/second, and tumor growth rate representation (obtained from the slope of the luciferase quantification plot). Note that only 2 out of 7 control animals survived at 27 days. Results are represented as mean ± SEM (*n* control arm = 2 and *n* TAT-Cx43_266–283_ = 5). Student’s *t*-test, *P value < .05. (D) Effect of TAT-Cx43_266–283_ on the survival of C57BL/6 mice bearing orthotopic tumor syngrafts. Percentage of animals alive along the experiment depicted in Kaplan–Meier plot (*n* control arm = 7 and *n* TAT-Cx43_266–283_ = 9 from at least 3 independent experiments). Log-rank test, *P value < .05. (E) Bright-field images showing hematoxylin–eosin staining of control and TAT-Cx43_266–283_-treated tumors, as well as magnifications of differences upon treatment in some GBM hallmarks like vascularization (left magnified images 40×), necrosis (middle magnified images 40×) and invasive borders (right magnified images 10×). Scale bar: 1 mm.

Among NSCs harboring EGFR alterations, NPE-IE cells have been reported to generate brain tumors in immunocompetent mice.^9^ Therefore, we selected NPE-IE cells to test the effect of TAT-Cx43_266–283_ on the development of tumors derived from NSCs harboring EGFR alterations and other GBM driver mutations. To compare these results with those previously obtained in the GL261-GSC GBM model,^36^ we followed the same treatment protocol, including dosage schedule and route of administration (Figure 5A). Briefly, TAT-Cx43_266–283_ was injected intracranially with tumor cells and, 7 days after the injection, TAT-Cx43_266–283_ was administered twice per week intraperitoneally. Bioluminescence imaging revealed a reduced tumor growth rate in those mice treated with TAT-Cx43_266–283_ (Figure 5B and C). In agreement with this, we found that TAT-Cx43_266–283_ treatment significantly enhanced the survival of mice with GBMs originating from NPE-IE cells (Figure 5D).

**Figure 6.**
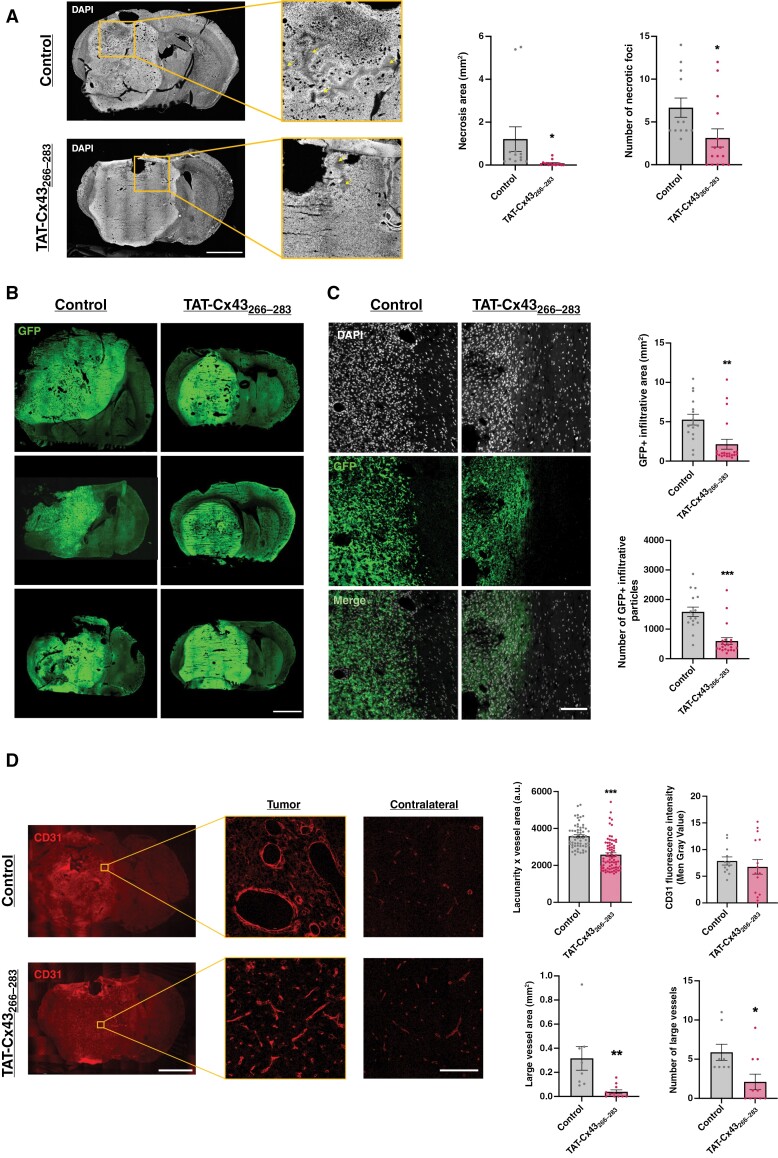
TAT-Cx43_266–283_ reduces necrosis formation, border invasiveness, and contributes to blood vessel normalization. (A) Confocal mosaic images depicting DAPI staining and the presence of necrotic foci ( arrows) in control vs TAT-Cx43_266–283_-treated animals, and quantification of the number and area of these necrotic foci. Brain sections were obtained from the animals when they showed humane endpoint signs. Results are expressed as the mean ± SEM of 3 independent experiments. At least 4 animals per experimental group were analyzed. Student’s *t*-test, *P value < .05 vs control. Scale bar: 1.9 mm. (B) Confocal mosaic images depicting GFP (endogenous marker of NPE-IE tumor cells) in whole-brain sections. Scale bar: 1.9 mm. (C) 63× confocal magnification images of tumor borders stained with DAPI and endogenously marked with GFP, and quantifications of border invasiveness measured as the number of GFP + particles invading from the tumor bulk and the area covered by these particles. Results are expressed as the mean ± SEM of 3 independent experiments. At least 4 animals per experimental group and 4 brain sections per animal were analyzed. Student’s *t*-test, **P value < .01, ***P value < .001 vs control. Scale bar: 100 μm. (D) CD31 immunofluorescence (vasculature marker) in brain sections of C57BL/6 mice bearing NPE-IE tumors and 63× magnifications of blood vessels in tumor and contralateral tissue in control and TAT-Cx43_266–283_-treated animals, as well as quantifications of vasculature-related parameters like CD31 intensity, blood vessel lacunarity (or heterogeneity degree) corrected by the CD31 area of the analyzed region, blood vessel area and number of large vessels in both experimental groups. Results are expressed as the mean ± SEM of 3 independent experiments. At least 4 animals per experimental group and at least 3 brain sections per animal were analyzed in all the experiments, and 4 tumor regions per brain slice were analyzed for lacunarity experiments. Student’s *t*-test, *P value < .05, **P value < .01, ***P value < .001. Scale bar: 1.9 mm. 63× magnification scale bar: 100 μm.

It has been previously shown that NPE-IE cells develop tumors that recapitulate hallmarks of human GBM in immunocompetent mice.^9^ Indeed, hematoxylin-eosin (H&E) stains of brain sections from endpoint mice showed that control mice developed aggressive tumors, which exhibited necrosis, infiltration, and high vascularization (Figure 5E). Interestingly, TAT-Cx43_266–283_-treated tumors showed a reduction in these hallmarks of human GBM (Figures 5E and 6). Thus, TAT-Cx43_266–283_ reduced the number and area of necrotic foci (Figure 6A and Supplementary Figure S13B). In addition, GFP images showed that TAT-Cx43_266–283_-treated tumors were more circumscribed in the brain parenchyma (Figure 6B and C). Indeed, the analysis of the infiltrative tumor borders indicated that TAT-Cx43_266–283_ reduced the number and area covered by GFP^+^ tumor cells invading the brain parenchyma (Figure 6C, and Supplementary Figure S13C and S14A). As H&E (Figure 5E) and whole-brain images (Supplementary Figure S13A) suggested differences in tumor vascularization, the levels of the vascular endothelial growth factor (VEGF) were analyzed. Immunohistochemistry and WB analyses showed that VEGF levels seemed to be reduced in TAT-Cx43_266–283_-treated tumors, although these results were not statistically significant (Supplementary Figure S15). Immunohistochemistry for the blood vessel marker, CD31, unveiled an interesting difference in tumor vessel morphology (Figure 6D). While control mice exhibited vessels of multiple sizes, some of them with a wide lumen, and aberrant morphologies, tumor vessels in TAT-Cx43_266–283_-treated animals were more uniform, smaller, and similar in shape to those found in the contralateral hemisphere (Figures 6D and Supplementary Figure S14B and C). These differences in CD31 pattern were analyzed by quantifying vessel lacunarity, a parameter that measures their degree of heterogeneity, which has been associated with shorter survival in patients.^41^ These analyses revealed that TAT-Cx43_266–283_-treated animals showed a significant decrease in lacunarity when compared to control animals (Figure 6D). Although the intensity of CD31 was not significantly modified by TAT-Cx43_266–283_, we found that the number of large blood vessels (with areas ≥ 0.1 mm^2^) and the area covered by them was significantly reduced by this treatment (Figure 6D and Supplementary Figure S14C).

## Discussion

Our previous studies showed that TAT-Cx43_266–283_ is a promising therapy against GBM because it exerts important antitumor effects in preclinical GBM models in vitro and in vivo.^35,36^ The results presented in this study represent an important step toward its clinical application. Indeed, we identified the frequent EGFR alterations in GBM as predictors of TAT-Cx43_266–283_ response and part of its mechanism of action, we showed effects in TMZ- and erlotinib-resistant GSCs and we revealed that TAT-Cx43_266–283_ targets NSCs with GBM-driver mutations, including EGFR alterations, in an immunocompetent GBM model in vivo.

The EGFR alterations found to be associated with TAT-Cx43_266–283_ effect in this study, such as EGFR amplification and EGFRvIII variant, are among the most common oncogenic^2,4^ and molecular hallmarks of GBM.^42^ Therefore, TAT-Cx43_266–283_ could potentially be beneficial to numerous GBM patients. In fact, this and previous studies showed that TAT-Cx43_266–283_ decreased cell viability in primary GSCs and GBM explants in most patients analyzed.^35^ Interestingly, although EGFR and EGFRvIII protein levels and activity match with TAT-Cx43_266–283_ effect, our results suggest that *EGFR* amplification or EGFRvIII version determined by genetic testing, commonly available in the clinical diagnostic, are sufficient to predict TAT-Cx43_266–283_ response. The lack of predictive response biomarkers hampers the success of many antitumor drugs that are successful in GBM preclinical studies. Therefore, we believe that the identification of EGFR alterations as biomarkers of TAT-Cx43_266–283_ response might help with patient stratification, which would be beneficial for the success of a future clinical trial.

EGFR-Src reciprocal activation loop is involved in cell transformation, and it is considered a prominent target for cancer therapy,^28,30^ including GBM.^5,31^ Indeed, exploring TCGA dataset, we found that EGFR alterations positively correlate with Src activity in IDHwt GBM patients. In addition, EGFR-altered patients showed reduced overall survival compared to those patients with unaltered EGFR, confirming the clinical relevance of the EGFR-Src axis in GBM patients. Importantly, our data suggest that TAT-Cx43_266–283_ targets this key EGFR-Src axis with the subsequent reduction in GSC survival. TAT-Cx43_266–283_ inhibits Src activity^34^ by acting as a docking platform for Src and its endogenous inhibitors CSK and PTEN.^33^ In this study, we found that TAT-Cx43_266–283_ targeted GSCs with PTEN deletion, suggesting that PTEN is not required for its effect. Phosphatases are not highly specific, therefore we speculated that this activity might be compensated by other phosphatases with the ability to inhibit Src, such as PTPN22.^43^ Although a direct effect of TAT-Cx43_266–283_ on EGFR cannot be excluded, TAT-Cx43_266–283_ might interfere in the EGFR-Src oncogenic loop by inhibiting Src activity, as Src phosphorylates and activates EGFR,^23,24,44^ which in turn triggers Src activity.^5,21,22^ In any case, the results obtained with EGFR- or EGFRvIII-engineered NSCs clearly demonstrated that EGFR or EGFRvIII overactivation was crucial for the reduction of Src activity and NSC viability promoted by TAT-Cx43_266–283_.

Previous studies showed that TAT-Cx43_266–283_ exhibits higher tumor cell selectivity and less toxicity than the Src inhibitor, dasatinib.^36,37^ Interestingly, in the human and mouse GSCs analyzed in the present study we found that the antitumor effect of TAT-Cx43_266–283_ was higher than that of TMZ, the standard treatment for GBM, and erlotinib, an inhibitor of EGFR with disappointing results in GBM clinical trials.^45^ In fact, so far EGFR inhibitors have not resulted in positive results in clinical trials, despite selecting GBM patients with specific EGFR alterations. However, the different mechanism of action of TAT-Cx43_266–283_, through the simultaneous targeting of Src, EGFR, and EGFRvIII, may represent an important advantage. In agreement with this, some altered NSCs that were resistant to TAT-Cx43_266–283_ and erlotinib showed a synergistic response to erlotinib plus TAT-Cx43_266–283_ combination. Although we cannot discard that erlotinib or TAT-Cx43_266–283_ targeted other signaling pathways responsible for this synergistic effect, perhaps the reduction in Src and EGFR activity, individually, was not sufficient to promote cell death in these NSCs, however when both kinases were inhibited cell survival was compromised. Src activity has been related to EGFR inhibitor-resistance,^46^ providing a rationale for the combination of EGFR and Src inhibitors, which has been studied in numerous clinical trials.^47,48^ Therefore, TAT-Cx43_266–283_ combination with new EGFR inhibitors deserves further exploration.

There is increasing evidence that some GBMs arise from NSCs with driver mutations.^8–10^ Interestingly, human GBM cells share critical mutations for GBM development with NSCs from the SVZ,^12^ which suggests that altered SVZ-NSCs might contribute to the usual tumor recurrence of most GBM patients. Therefore, targeting SVZ-NSCs with GBM driver mutations represents a promising strategy to prevent GBM recurrence. Importantly, TAT-Cx43_266–283_ reduced the viability of SVZ-NSCs with GBM driver mutations. In fact, TAT-Cx43_266–283_ reduced the rate of tumor growth, enhancing the survival of immunocompetent mice with GBM originated from NPE-IE, which are SVZ-NSCs with GBM-driver mutations and immune evasive properties. Immunohistology performed in postmortem mice brain tissue demonstrated that TAT-Cx43_266–283_ reduced important hallmarks of GBMs, such as infiltrative borders, necrotic areas, and aberrant vasculature, indicating that TAT-Cx43_266–283_ reduces the malignancy grade of these tumors. Together, these results highlight the potential benefit of TAT-Cx43_266–283_ for GBM recurrence.

We previously reported that TAT-Cx43_266–283_ reduces GSC invasion in vitro^35^ and in vivo^36^ through Src-FAK axis, which may be the mechanism underlying the reduction of tumor infiltration found in the present study in TAT-Cx43_266–283_-treated GBMs originated by SVZ-NSCs with EGFR alterations and GBM driver mutations. In addition, EGFR and Src have been involved in VEGF secretion leading to angiogenic tumor growth.^5,49^ Therefore, TAT-Cx43_266–283_ might reduce the aberrant vascularization found in GBMs, originated by SVZ-NSCs with GBM driver mutations, through the inhibition of the EGFR-Src axis. In addition to expanding our knowledge about the mechanism of action of TAT-Cx43_266–283_, by including a pivotal role of EGFR, this study supports the reported relevance of EGFR-Src axis to drive the aggressive nature of GBMs by promoting invasion and angiogenesis.^5^

In conclusion, although additional preclinical studies are required, including optimal dose, schedule, and route of administration, as well as the study of combination of TAT-Cx43_266–283_ with other therapeutic strategies, our results further support the clinical application of TAT-Cx43_266–283_ against GBM.

## Supplementary material

Supplementary material is available online at *Neuro-Oncology* (https://academic.oup.com/neuro-oncology).

noae060_suppl_Supplementary_Materials

## Data Availability

All data generated in this study are available upon reasonable request from the corresponding author, Prof. Arantxa Tabernero (ataber@usal.es).
