# Supplementary material for: EGFR amplification and EGFRvIII predict and participate in TAT-Cx43266–283 antitumor response in preclinical glioblastoma models
Source: Neuro Oncol. 2024 Mar 20;26(7):1230–46. doi: 10.1093/neuonc/noae060 (PMC11226870; doi:10.1093/neuonc/noae060)
Supplement: noae060_suppl_Supplementary_Materials [file noae060_suppl_supplementary_materials.zip › Supplementary material/Supplementary information clean.docx]

**Supplementary information**

**Supplementary methods**

**Animals**

Equal number of male and female C57BL/6 mice were obtained from Charles River Laboratories and the animal facility of the University of Salamanca. Mice were housed individually in cages provided with environmental enrichment and food and water *ad libitum*, in a mouse room with a 12-hour light/12-hour dark light cycle. Animal procedures were approved by the ethics committee of the University of Salamanca and the Junta de Castilla y León (Spain) and were carried out in accordance with European Community Council directives (2010/63/UE), and Spanish law (RD 53/2013 BOE 34/11370–420, 2013) for the use and care of laboratory animals.

**Cell culture details**SVZ-NSCs, SVZ-EGFRwt and SVZ-EGFRvIII murine cell lines were cultured in DMEM/F-12 Glutamax medium (Gibco) supplemented with a mix of antibiotics and antimycotics [0.064 mg/mL penicillin G (Sigma, ref: P3032-10MU), 1 mg/mL streptomycin (Sigma, ref: S9137-25G) and 0.46 μg/mL amphotericin B (Sigma, ref: A9528-50MG)], as well as 1% B27 (Gibco, ref: 17504044), 0.5% N2 (Gibco, ref: 17502048), 20 ng/mL human EGF (Peprotech, ref: #AF-100-15) and 20 ng/mL human b-FGF (Invitrogen, ref: RP8628; Peprotech, ref: #100-18B).

NP, NPE and NPE-IE cell lines express GFP and luciferase as reporters. NSCs-EGFRvIII are NSCs with EGFRvIII overexpression, NP are NSCs with CRISPR/Cas-mediated ablation of Nf1 and PTEN, NPE are NSCs with CRISPR/Cas-mediated ablation of Nf1 and PTEN and EGFRvIII overexpression, and NPE-IE are NSCs with CRISPR/Cas-mediated ablation of Nf1 and PTEN and EGFRvIII overexpression together with immune evasive properties developed after several intracranial transplantations in immunocompetent mice ^1^. Patient-derived GSC lines L0, L1 and L2 were obtained from the University of Florida and have been previously described^2^. T4121 cells were obtained from Duke University and have been described previously^3^. DI318 cells were obtained from Cleveland Clinic and have been previously described^4^. 23M cells were obtained from University of Texas/MD Anderson Cancer Center and have been previously described^5^, and control immortalized hNSC were obtained from Fred Hutchinson Cancer Center/University of Washington and have been previously described^6^. These cell lines were cultured in GSC medium: DMEM/F-12 with glutamine (Sigma) supplemented with 1.45% glucose (Sigma, ref: G8644), 1% MEM NEAA 100X (Gibco), 1% Penicillin-Streptomycin (Gibco, ref: 15140-122), 0.16% BSA (Gibco, ref: 15260-037), 0.2% β-mercaptoethanol (Gibco), 1% B27, 0.5% N2, 10 ng/mL human EGF and 10 ng/mL human b-FGF. Either 4 μg/mL laminin (R&D Systems) or 0.06% Geltrex (Thermo Fisher) was added to the media, and TC-treated surfaces were used to ensure cell adherence. Additionally, E20, E26 and E51 lines were cultured in surfaces pre-coated with 10 ug/mL laminin (Invitrogen) for at least 1 hour before use.

Cells were grown until they were confluent, and Accutase (Corning) was used for cell dissociation during passages. Cells were split according to the needs of the experiment and counted using a Countess Automatic Cell Counter (Invitrogen). All cell lines were cultured in an incubator with an atmosphere of 37°C and 5% CO_2_. All cell lines were mycoplasma-free, and routinely tested for Mycoplasma spp. by PCR using a Mycoplasma Gel Form kit (Biotools).

**Treatments**

Lyophilized peptides (>95% pure) were obtained from GenScript. YGRKKRRQRRR was used as the TAT sequence.

For in vitro experiments, the peptides were dissolved in DMEM/F-12 medium and used at 50 μM in culture medium. Unless otherwise stated in the figure legends, temozolomide (TMZ, MedChemExpress, ref: HY-17364 or Sigma, ref: T2577-100MG) was dissolved in DMEM/F-12 medium and used at 100 μM in culture medium. Erlotinib (Sigma) was dissolved in filtered DMSO and used at 1, 5 and 10 μM in culture medium. 0.05%, 0.1% and 0.2% (v/v) DMSO was used as an erlotinib vehicle control. All treatments were added to the culture medium either once (24 hours after plating the cells) for protein extraction, or twice (24 and 96 hours after plating the cells) for cell viability experiments. For Matrigel invasion assays, TAT-Cx43_266–283_ was added directly to the cells as they were plated.

For in vivo experiments, an intracranial injection of 2 μL containing 200,000 GSCs and 100 μM TAT-Cx43_266–283_ in GSC medium, or the equivalent volume of GSC medium, was performed. One week after the injection of tumor cells, either 4 nmol/g TAT-Cx43_266–283_ in 0.9% NaCl or the equivalent volume of 0.9% NaCl was intraperitoneally injected twice a week for the duration of the experiment.

**Alamar blue viability assay**

Cells were incubated with 10% Alamar blue/resazurin (Bio-Techne R&D Systems or Bio-Rad) for 6 hours, and fluorescence was measured using an Appliskan microplate reader (λ_ex_ = 560 nm and λ_em_ = 590 nm).

**Matrigel invasion assay**

Matrigel invasion assays were performed as previously described. Briefly, 8 µm-pore PET inserts (Falcon, ref: 353097) were placed in Falcon companion 24-well plates (ref: 353504), and they were coated with 100 µL of 0.25 mg/mL Matrigel matrix (Corning, ref: 354234) diluted in DMEM/F12 supplemented with 1.45% glucose (Sigma, ref: G8644), 1% MEM NEAA 100X (Gibco), 1% Penicillin-Streptomycin (Gibco, ref: 15140-122), 0.16% BSA (Gibco, ref: 15260-037) and 0.2% β-mercaptoethanol (Gibco). Plates with coated inserts were incubated at 37ºC for approximately 2 hours, as recommended by the manufacturer. Before plating the cells on the insert, the remaining Matrigel coating buffer was removed. Next, 75,000 cells in 200 µL of GSC medium, together with the different treatments, were plated in the upper chamber. Finally, 500 µL of DMEM/F12 + 10% FBS were added to the lower well and cells were allowed to invade for 15 hours. After that period of time, cells in the upper part of the insert were carefully removed with cotton swabs. The invading cells on the lower part of the insert were fixed with 4% paraformaldehyde for 10 min, washed with PBS, and stained with 1:20 Giemsa (Sigma, ref: for 10 min. The inserts were washed by several immersions in distilled water and allowed to air dry. Bright-field images of the inserts were acquired using a Zeiss SteREO Discovery.V8 stereomicroscope (8x zoom) coupled to an AxioCam ICc3 camera, and cells were counted manually using Fiji software. At least 2 random fields per Matrigel insert were analyzed.

**Time-lapse microscopy for the analysis of cell migration**

For migration experiments, 5,000 cells/well were plated in 24-well plates. The following day, 50 µM TAT-Cx43_266-283_ was added and, approximately 1 hour after adding the treatments, cells were recorded using the time-lapse live cell imaging microscope Zeiss Axio Observer Z1 coupled to an AxioCam MRm camera, which acquired phase-contrast photographs of the cells every 10 minutes for at least 24 hours. Videos were exported at 10 frames per second using Zen imaging software. Cells were tracked in at least 20 frames per video and their mean velocity was obtained using Fiji software. Approximately 5 cells were tracked per video. The velocity of the cell lines was analyzed in frames corresponding to 20 hours after the addition of the treatment, except in GSC lines E26 and E43, which were analyzed 6.7 hours after the addition of the treatment due to the quick cell death that is observed in these lines after treatment.

**Detailed in vivo procedures**

The murine GSC line NPE-IE was intracranially injected into the brains of 8-week-old C57BL/6 mice as previously described ^1^. Briefly, mice were anaesthetized with a mixture of 1.5-2% isoflurane and oxygen at a 1 L/min flow rate and placed in a stereotaxic frame. The skull was trephined according to the stereotaxic coordinates, and 2 μL of GSC medium containing 200,000 cells were injected in the right striatum using a 26S-needle Hamilton microsyringe. Coordinates were +0.5 mm AP, +1.5 LM to bregma, and -2.5 mm deep. Before injecting at the described depth, a small pocket for the cells was formed by introducing the needle to a depth of -3.5 mm. The cell suspension was injected slowly, and the needle was held in place for 2 min after cell injection. Cells were kept on ice while the surgery was being performed.

Animals were monitored daily during the first week after surgical procedures, and at least twice a week for the rest of the experimental period. Animals showing signs of humane endpoint (briefly, a decrease of 15-20% in their body weight, piloerection, hunched posture, lethargy or hyperexcitability) were sacrificed. An additional endpoint criterion was tumor size, indicated by luciferase activity of tumor cells.

Bioluminiscence imaging was performed approximately every two weeks after the implantation of the cells. Briefly, mice were intraperitoneally injected with 150 μg/g D-Luciferin (Goldbio). Then, mice were anaesthetized with a mixture of 3% isoflurane and oxygen at a 1 L/min flow rate and, 10 minutes after the luciferin injection, they were imaged using an IVIS Lumina S5 (Perkin Elmer) for 5, 10, 30, 60 and 120 seconds of exposure. Binning parameters depended on the features (bioluminescence and size) of the tumor. For luciferase activity analysis, the software Living Image (Perkin Elmer) was used, and the same ROI was used for all the images. All exposure times were quantified, and the highest signal (measured in photons/s or p/s) was selected as the measure of luciferase activity for every mouse. This measure was used as an endpoint criterion if it was over 8.10^8^ p/s, as it is an indicator of the tumor size.

When mice met the established endpoint criteria, they were intraperitoneally injected with 120 mg/kg sodium pentobarbital (Vetoquinol) and, upon loss of toe pinch reflex, they were transcardially perfused at a 5.5 mL/min flow rate with 27.5 mL 0.9% NaCl and 55 mL 4% PFA. Brains were extracted and kept in 4% PFA overnight, and then transferred to a cryoprotectant 30% sucrose in PBS solution until they sank. Then, brains were sliced to obtain 20 and 40 μm coronal sections using a Microm HM550 cryostat.

**Detailed immunofluorescence protocol**

For CD31 immunofluorescence, brain sections were washed with PBS with 0.1% Triton X-100 and blocked at 4°C overnight with a blocking solution (PBS with 1% Triton X-100 and 10% donkey serum). Then, sections were incubated at 4°C overnight with a rat anti-CD31 monoclonal antibody (1:100, BD Biosciences, ref: 550274). Next, sections were washed with PBS with 0.1% Triton X-100 and incubated at 4°C overnight with a goat anti-rat IgG Alexa Fluor A594-conjugated secondary antibody (1:500, Invitrogen, ref: #A-11007). Finally, nuclei were stained with DAPI (1:5000, Invitrogen) for 5 min and sections were mounted using SlowFade™ Gold antifade mountant (Thermo Fisher).

For VEGF immunofluorescence, brain sections were washed with PBS with 0.1% Tween and blocked for 2 hours with a blocking solution (PBS with 0.1% Tween, 10% goat serum and 5% BSA). Then, sections were incubated at 4°C overnight with a mouse anti-VEGF monoclonal antibody (1:50, Santa Cruz Biotechnology, ref: sc-7269). Next, sections were washed with PBS with 0.1% Tween and incubated for 2 hours r.t. with a goat anti-mouse IgG Alexa Fluor A594-conjugated secondary antibody (1:500, Invitrogen, ref: #A-11032). Finally, nuclei were stained with DAPI (1:5000, Invitrogen) for 5 min and mounted as described.

Mosaic images of the sections were acquired using a Leica Stellaris 8 confocal microscope, selecting only one plane of the z axis and using a 10X objective and a 63X oil objective. Images were processed and analyzed using the softwares LAS X (Leica) and Fiji.

**Blood vessel analyses**

Blood vessel lacunarity was measured in at least four regions per animal from raw CD31 confocal mosaic images. Mean lacunarity and vessel area (i.e., CD31 area) were analyzed in these regions using the software AngioTool 0.6a (N.C.I., U.S.A.). Briefly, blood vessels were selected according to their intensity, non-specific small particles were removed and mean lacunarity was measured. Due to possible differences in mean lacunarity caused by a difference in the number of vessels in each region, values of vessel area and mean lacunarity were used to calculate a corrected lacunarity value (mean lacunarity x vessel area).

The number and area of large blood vessels (areas ≥ 0.1 mm^2^) were quantified in CD31 confocal mosaic images by manually selecting the vessel area using Fiji.

**Estimation of the invasiveness of intracranial tumors**

The invasiveness of GFP+ NPE-IE cells was determined using Fiji software by thresholding GFP fluorescence images of the whole coronal brain section, establishing the tumor rims, and quantifying the area occupied by GFP+ NPE-IE cells infiltrating the brain parenchyma from the main tumor bulk.

**Hematoxylin-eosin (H&E) staining**

H&E staining was performed by the Compared Molecular Pathology service of Centre for Cancer Research (IBMCC, Salamanca). Bright-field images were acquired using an Olympus BX51 microscope coupled to an Olympus DP74 camera, and the software CellSens Entry 4.1.1.

**Fluorescence In Situ Hybridization (FISH)**

For FISH studies to analyze EGFR amplification in human GSC, 10^6^ cells per GSC line were used. The Cytometry Service of the University of Salamanca performed FISH experiments using a EGFR/CEP7 probe (Metasystems).

**RNA-seq**

For RNA-seq analyses, 10^6^ cells per cell line (L0, L1, L2, T4121, DI318, 23M and hNSC) were used. RNA was extracted by the DNA National Bank (University of Salamanca) using organic solvents (phenol-chloroform). The Sequencing Service (University of Salamanca) generated RNA libraries using a KAPA-mRNA HyperPrep-Kit for Illumina platforms (Roche). Sequencing was performed using a NovaSeq 6000 (Illumina).

EGFRvIII status was analyzed by aligning sequencing data to the human genome, and Sashimi plots were obtained using the software IGV (U.C. San Diego).

**Detailed western blot protocol**

WB were performed as previously described. Briefly, for cell protein extraction, 100,000 cells were plated in 35 mm diameter dishes, and treated as described. 24 hours after the first dose of treatment, proteins were extracted using an extraction buffer (2% SDS, 2 mMM EDTA, 5 mM TRIS-HCl pH 6.8 and 2 mM EGTA in ddH_2_O) with 1:100 protease (Fisher Scientific) and phosphatase inhibitor cocktails (Fisher Scientific). For tumor tissue protein extraction, two 40 μm coronal brain sections were used. The tumor area was isolated and transferred to an Eppendorf containing 60 μL of protein extraction buffer with 1:100 protease and phosphatase inhibitor cocktails as described above. Samples were heated at 99°C for 5 min and sonicated for 5 min. Loading buffer 1X (Thermo Fisher) and reducing agent 1X (Thermo Fisher) were added to the samples and heated at 70°C for 10 min. Between 16-22 μg of proteins were loaded and separated on NuPaGE Novex Bis-Tris 4-12% midi gels (Invitrogen) at room temperature and 120V. Proteins were transferred to iBlot nitrocellulose membranes (Invitrogen) using an iBlot dry blotting system (Invitrogen). Ponceau staining (Sigma) was used to observe total protein load. Membranes were cut into strips to incubate them with different antibodies, allowing a comparative analysis of the amount of each protein in the same sample. Membranes were blocked with 5% dry milk and incubated overnight at 4°C with primary antibodies: anti-phospho-EGFR Y1068 (1:1000, Cell Signaling, ref: 3777) and anti-EGFR (1:1000, Cell Signaling, ref: 4267), anti-phospho-Src Y416 (1:250, Cell Signaling, ref: 2101S) and anti-Src (1:500, Cell Signaling, ref: 2110S), anti-VEGF (1:200, Santa Cruz Biotechnology, ref: sc-7269), anti-GAPDH (1:5000, Invitrogen, ref: AM4300) and anti-β-actin (1:1000, Sigma, ref: A5441). Then, membranes were washed and incubated with HRP-conjugated anti-rabbit IgG (1:2500, Santa Cruz Biotechnology, ref: sc-2357-CM) or HRP-conjugated anti-mouse IgG (1:5000, Jackson ImmunoResearch, ref: 115-035-003), and developed using luminol (Santa Cruz Biotechnology) and a MicroChemi imaging system (Bioimaging Systems). Replicate blots are included in the Supplementary Figures.

**Supplementary references**

**1.** Gangoso E, Southgate B, Bradley L, et al. Glioblastomas acquire myeloid-affiliated transcriptional programs via epigenetic immunoediting to elicit immune evasion. *Cell.* 2021; 184(9):2454-2470.e2426.

**2.** Deleyrolle LP, Harding A, Cato K, et al. Evidence for label-retaining tumour-initiating cells in human glioblastoma. *Brain.* 2011; 134(Pt 5):1331-1343.

**3.** Lathia JD, Gallagher J, Myers JT, et al. Direct in vivo evidence for tumor propagation by glioblastoma cancer stem cells. *PLoS One.* 2011; 6(9):e24807.

**4.** Sundar SJ, Shakya S, Barnett A, et al. Three-dimensional organoid culture unveils resistance to clinical therapies in adult and pediatric glioblastoma. *Transl Oncol.* 2022; 15(1):101251.

**5.** Bhat KPL, Balasubramaniyan V, Vaillant B, et al. Mesenchymal differentiation mediated by NF-κB promotes radiation resistance in glioblastoma. *Cancer Cell.* 2013; 24(3):331-346.

**6.** Hubert CG, Bradley RK, Ding Y, et al. Genome-wide RNAi screens in human brain tumor isolates reveal a novel viability requirement for PHF5A. *Genes Dev.* 2013; 27(9):1032-1045.

**Supplementary figure legends**

**Supplementary Fig 1. WB of active and total EGFR and EGFRvIII in patient-derived GSCs.** Related to Fig. 1. WB replicates from Fig. 1B.

**Supplementary Fig 2**. **WB quantifications of active and total EGFR and EGFRvIII in control and TAT-Cx43_266-283_-treated patient-derived GSCs**. Related to Fig 1. **(A)** WB quantifications of total and active EGFR and EGFRvIII levels in human patient-derived GSCs and hNSCs. β-actin was used to normalize protein values among cells. **(B)** WB quantification of the effect of TAT-Cx43_266-283_ in the levels and activity of EGFR and EGFRvIII. Student’s t-test, * p value < 0.05, ** p value < 0.01, *** p value < 0.001 vs control.

**Supplementary Fig 3**. **Characterization of EGFR amplification and correlation graph in patient-derived GSCs.** Related to Fig. 1. **(A)** FISH images of a subset of patient-derived GSC lines. EGFR probe in red and CEP7 probe in green. Images were acquired using a 100X objective. **(B)** Summary table of EGFR status of all patient-derived cell lines according to RNAseq and FISH studies. **(C)** Correlation graph depicting the relationship between EGFR alterations in GSCs (according to their total EGFR score) and their response to TAT-Cx43_266-283._ **(D)** Summary table of the IDH and MGMT status and recurrecence information of all patient-derived GSCs.

**Supplementary Fig 4. Sashimi plots of patient-derived GSCs and hNSCs**. Related to Fig. 1. Sashimi plots representing the number of reads of EGFR exons and junctions in human cell lines. Exons 2-7, lost in EGFRvIII version of EGFR, are marked.

**Supplementary Fig 5. Growth curves of patient-derived GSCs in response to different treatment.** Related to Fig. 2. **(A-E)** Representative Phase-contrast images and growth curves of patient-derived GSCs with [E28 (A), E22 (B), E26 (C), and E43 (D)] and without [E20 (E)] EGFR alterations, obtained by Alamar blue assays 24, 48, 72 and 144 hours after the first dose of the treatments described in Fig. 2. Scale bar: 100 µm

**Supplementary Fig 6. Erlotinib dose-response data and comparison with TAT-Cx43_266-283_ effect in cell viability.** Related to Fig. 2. Patient-derived GSCs were treated with 1, 5 and 10 μM erlotinib or DMSO (v/v). **(A)** Alamar blue viability assay after 6 days of treatments administered at days 0 and 3. Results are represented as mean ± S.E.M. of 3 independent experiments. ANOVA, * p value < 0.05, ** p value < 0.01, *** p value < 0.001 versus control. **(B)** Alamar blue viability assay of 10 μM erlotinib versus TAT-Cx43_266-283._ Results are represented as mean ± S.E.M. of 3 independent experiments. ANOVA, * p value < 0.05, ** p value < 0.01, *** p value < 0.001 versus control.

**Supplementary Fig 7**. **WB replicates and quantifications of active and total EGFR and EGFRvIII in control and TAT-Cx43_266-283_-treated SVZ-NCSs with or without EGFR alterations.** Related to Fig. 3**. (A)** WB replicates from Fig. 3C. **(B)** WB quantifications of total and active EGFR and EGFRvIII levels in normal and EGFR altered SVZ-NSCs. GAPDH was used to normalize protein values among cells. **(C)** WB quantification of the effect of TAT-Cx43_266-283_ in the levels and activity of EGFR and EGFRvIII in these lines. Student’s t-test, *** p value < 0.001 vs control.

**Supplementary Fig 8**. **WB replicates and quantifications of active and total Src in control and TAT-Cx43_266-283_-treated SVZ-NCSs with or without EGFR alterations.** Related to Fig. 3. **(A)** WB replicates of active (Y416 phosphorylation) and total Src in control and treated murine GSCs from the SVZ **(B)** WB quantification of the effect of TAT-Cx43_266-283_ in the levels and activity of Src in these lines. Protein values were normalized using GAPDH. Student’s t-test, *** p value < 0.001 vs control.

**Supplementary Fig 9. Growth curves of murine NSCs with GBM-driver mutations in response to different treatments.** Related to Fig. 3. **(A-C)** Representative phase-contrast images and growth curves of murine NSC-EGFRvIII (A), NP (B) and NPE (C) lines obtained by Alamar blue assays 24, 48, 72 and 144 hours after the first dose of the treatments described in Fig. 3. Scale bar: 100 µm. **(D)** WB replicates from Fig. 3F.

**Supplementary Fig 10**. **WB replicates and quantifications of active and total EGFR and EGFRvIII in control and TAT-Cx43_266-283_-treated NSCs with GBM-driver mutations.** Related to Fig 3. **(A)** WB quantifications of total and active EGFR and EGFRvIII levels in murine NSCs with glioma-driver alterations. β-actin was used to normalize protein values among cells. **(B)** WB quantification of the effect of TAT-Cx43_266-283_ in the activation and levels of EGFR and EGFRvIII in these lines. Results are expressed as the mean ± S.E.M. of 3 independent experiments. Student’s t-test, * p value < 0.05, *** p value < 0.001 vs control.

**Supplementary Fig 11. WB quantification of active and total Src levels in control and TAT-Cx43_266-283_-treated NSCs with GBM-driver mutations and TMZ dose-response representation in murine SVZ-NSCs with and without EGFR alterations.** Related to Fig 3. (A) WB quantification of the effect of TAT-Cx43_266-283_ in the levels and activity of Src in these lines. Protein values were normalized using β-actin. Results are expressed as the mean ± S.E.M. of 3 independent experiments. Student’s t-test, * p value < 0.05, *** p value < 0.001 vs control. **(B)** Heatmap summarizing EGFR alterations of murine GSCs and hNSCs determined by WB. For total color score, every square was assigned a numeric value according to its color. Dark red = 1 point, light red = 0.75 points, yellow = 0.5 points and green = 0 points. **(C)** Alamar blue viability assay of murine SVZ-NSCs with and without EGFR alterations treated with 25, 50, 100 and 200 μM TMZ. TAT-Cx43_266-283_ treatment is represented as a red dashed line. Results are expressed as the mean ± S.E.M. of 3 independent experiments. ANOVA test was performed for statistical analysis. **(D)** Alamar blue viability assay of murine SVZ-NSCs with and without EGFR alterations treated as described in Fig 3A, but comparing all treatments with the highest TMZ dose administered (200 μM). Note that this high TMZ dose exerts a more potent effect in the cell viability of SVZ-EGFRvIII cells but also has an effect in the cell viability of control healthy SVZ-NSCs. Results are expressed as the mean ± S.E.M. of at least 3 independent experiments. ANOVA, * p value < 0.05, *** p value < 0.001 vs control.

**Supplementary Fig 12.** **WB replicates and quantifications of active and total Src in control and TAT-Cx43_266-283_-treated patient-derived GSCs.** Related to Fig. 3. **(A)** WB replicates of active (Y416 phosphorylation) and total Src in control and treated patient-derived GSCs **(B)** WB quantification of the effect of TAT-Cx43_266-283_ in the levels and activity of Src in these lines. Protein values were normalized using GAPDH. Results are expressed as the mean ± S.E.M. of 2 or 3 independent experiments (depending on the cell line, see individual dots in the graph). Student’s t-test, * p value < 0.05, ** p value < 0.01 vs control.

**Supplementary Fig 13. Whole brain images from the analyzed animals, DAPI images for necrosis analysis and GFP invasive borders analysis pipeline.** Related to Fig. 6. **(A)** Images of brains extracted from endpoint C57BL/6 animals with NPE-IE tumors treated or not with TAT-Cx43_266-283._ **(B)** Replicates of confocal mosaic images of DAPI immunofluorescence to analyze the number and area of necrotic foci described in Fig 6A. Each brain section represents a different animal. Scale bar: 2 mm. **(C)** Scheme of the GFP invasive border analysis Fiji pipeline depicting the selection of the tumor borders by thresholding GFP signal, and the quantification of invading particles. Scale bar: 1.9 mm. Magnification scale bar (yellow line): 210 μm.

**Supplementary Fig 14. Replicates of GFP and CD31 immunofluorescence.** Related to Fig 6. **(A)** Replicates of 63X confocal images of GFP invading cells in control and treated animals. Each column within both experimental groups represents a different animal. Scale bar: 100 μm. **(B)** Replicates of confocal mosaic images of brain sections immunostained with CD31 in control and treated animals. Each brain section represents a different animal. Scale bar: 1.7 mm. **(C)** Replicates of 63X confocal images of tumor vasculature immunostained with CD31. Each image represents the vasculature of a different animal. Scale bar: 100 μm.

**Supplementary Fig 15. Analysis of VEGF levels in tumor tissue of control and TAT-Cx43_266-283_-treated animals.** Brains of endpoint animals were used for obtaining 40 μm brain sections for immunofluorescence studies and protein extraction for western blot. **(A)** Analysis of VEGF fluorescence intensity of confocal mosaic images of control and TAT-Cx43_266-283_-treated animals. Three animals per experimental group and 5 tumor border regions were analyzed in each animal. Results are expressed as the mean ± S.E.M. of 3 animals per experimental group in which a single brain section was analyzed. Student’s t-test was performed to assess statistical significance. **(B)** WB images depicting levels of VEGF in tumor tissue of control and treated mice. β-actin was used as a loading control.

**Supplementary Table 1.** List of proteins that differed among patients with or without EGFR alterations. Data was downloaded from the TCGA GBM database using cBioportal.

**Supplementary Movie 1. Time lapse movie of untreated E15 patient-derived GSCs.** Phase-contrast time-lapse movie of E15 GSCs 20 hours after starting the experiment. Images were acquired at 10 min intervals as described in the Experimental procedures section. Frame size= 447.63 x 335.4 μm. Frame rate= 10 frames per second.

**Supplementary Movie 2. Time lapse movie of TAT-Cx43_266-283_-treated E15 patient-derived GSCs.** Phase-contrast time-lapse movies of E15 GSCs 20 hours after adding 50 μM TAT-Cx43_266-283_ and starting the experiment. Images were acquired at 10 min intervals as described in the Experimental procedures section. Frame size= 447.63 x 335.4 μm. Frame rate= 10 frames per second.

**Supplementary Movie 3. Time lapse movie of untreated E20 patient-derived GSCs.** Phase-contrast time-lapse movie of E20 GSCs 20 hours after starting the experiment. Images were acquired at 10 min intervals as described in the Experimental procedures section. Frame size= 447.63 x 335.4 μm. Frame rate= 10 frames per second.

**Supplementary Movie 4. Time lapse movie of TAT-Cx43_266-283_-treated E20 patient-derived GSCs.** Phase-contrast time-lapse movies of E20 GSCs 20 hours after adding 50 μM TAT-Cx43_266-283_ and starting the experiment. Images were acquired at 10 min intervals as described in the Experimental procedures section. Frame size= 447.63 x 335.4 μm. Frame rate= 10 frames per second.

**Supplementary Movie 5. Time lapse movie of untreated E22 patient-derived GSCs.** Phase-contrast time-lapse movie of E22 GSCs 20 hours after starting the experiment. Images were acquired at 10 min intervals as described in the Experimental procedures section. Frame size= 447.63 x 335.4 μm. Frame rate= 10 frames per second.

**Supplementary Movie 6. Time lapse movie of TAT-Cx43_266-283_-treated E22 patient-derived GSCs.** Phase-contrast time-lapse movies of E22 GSCs 20 hours after adding 50 μM TAT-Cx43_266-283_ and starting the experiment. Images were acquired at 10 min intervals as described in the Experimental procedures section. Frame size= 447.63 x 335.4 μm. Frame rate= 10 frames per second.

**Supplementary Movie 7. Time lapse movie of untreated E26 patient-derived GSCs.** Phase-contrast time-lapse movie of E26 GSCs 6.7 hours after starting the experiment. Images were acquired at 10 min intervals as described in the Experimental procedures section. Frame size= 447.63 x 335.4 μm. Frame rate= 10 frames per second.

**Supplementary Movie 8. Time lapse movie of TAT-Cx43_266-283_-treated E26 patient-derived GSCs.** Phase-contrast time-lapse movies of E26 GSCs 6.7 hours after adding 50 μM TAT-Cx43_266-283_ and starting the experiment. Images were acquired at 10 min intervals as described in the Experimental procedures section. Frame size= 447.63 x 335.4 μm. Frame rate= 10 frames per second.

**Supplementary Movie 9. Time lapse movie of untreated E28 patient-derived GSCs.** Phase-contrast time-lapse movie of E28 GSCs 20 hours after starting the experiment. Images were acquired at 10 min intervals as described in the Experimental procedures section. Frame size= 447.63 x 335.4 μm. Frame rate= 10 frames per second.

**Supplementary Movie 10. Time lapse movie of TAT-Cx43_266-283_-treated E28 patient-derived GSCs.** Phase-contrast time-lapse movies of E28 GSCs 20 hours after adding 50 μM TAT-Cx43_266-283_ and starting the experiment. Images were acquired at 10 min intervals as described in the Experimental procedures section. Frame size= 447.63 x 335.4 μm. Frame rate= 10 frames per second.

**Supplementary Movie 11. Time lapse movie of untreated E43 patient-derived GSCs.** Phase-contrast time-lapse movie of E43 GSCs 6.7 hours after starting the experiment. Images were acquired at 10 min intervals as described in the Experimental procedures section. Frame size= 447.63 x 335.4 μm. Frame rate= 10 frames per second.

**Supplementary Movie 12. Time lapse movie of TAT-Cx43_266-283_-treated E43 patient-derived GSCs.** Phase-contrast time-lapse movies of E43 GSCs 6.7 hours after adding 50 μM TAT-Cx43_266-283_ and starting the experiment. Images were acquired at 10 min intervals as described in the Experimental procedures section. Frame size= 447.63 x 335.4 μm. Frame rate= 10 frames per second.

**Supplementary Movie 13. Time lapse movie of untreated E51 patient-derived GSCs.** Phase-contrast time-lapse movie of E51 GSCs 20 hours after starting the experiment. Images were acquired at 10 min intervals as described in the Experimental procedures section. Frame size= 447.63 x 335.4 μm. Frame rate= 10 frames per second.

**Supplementary Movie 14. Time lapse movie of TAT-Cx43_266-283_-treated E51 patient-derived GSCs.** Phase-contrast time-lapse movies of E51 GSCs 20 hours after adding 50 μM TAT-Cx43_266-283_ and starting the experiment. Images were acquired at 10 min intervals as described in the Experimental procedures section. Frame size= 447.63 x 335.4 μm. Frame rate= 10 frames per second.
