# Supplementary figures and images for: EGFR amplification and EGFRvIII predict and participate in TAT-Cx43266–283 antitumor response in preclinical glioblastoma models
Source: Neuro Oncol. 2024 Mar 20;26(7):1230–46. doi: 10.1093/neuonc/noae060 (PMC11226870; doi:10.1093/neuonc/noae060)

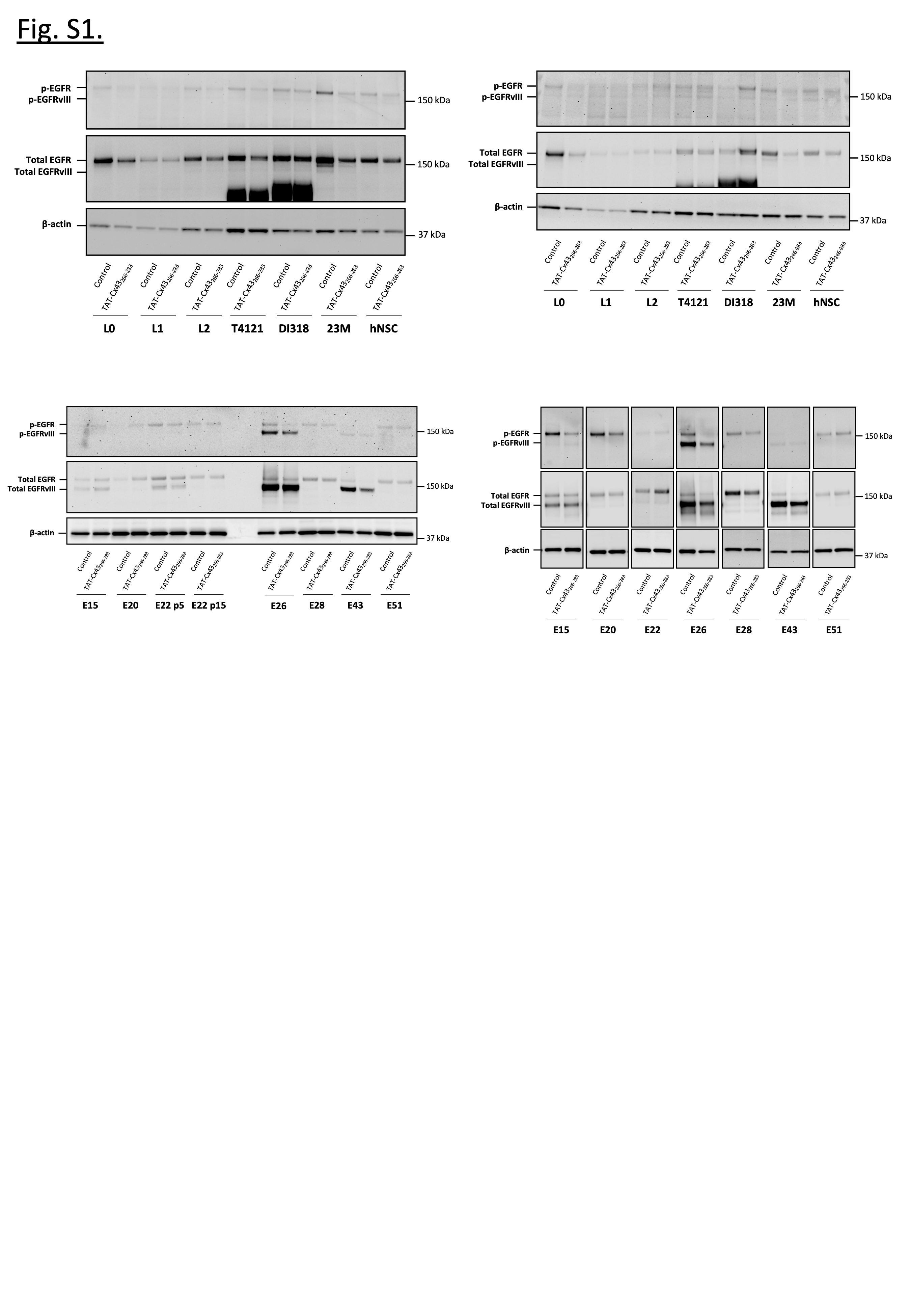

Supplement: noae060_suppl_Supplementary_Materials [file noae060_suppl_supplementary_materials.zip › Supplementary material/FigS1.tiff]

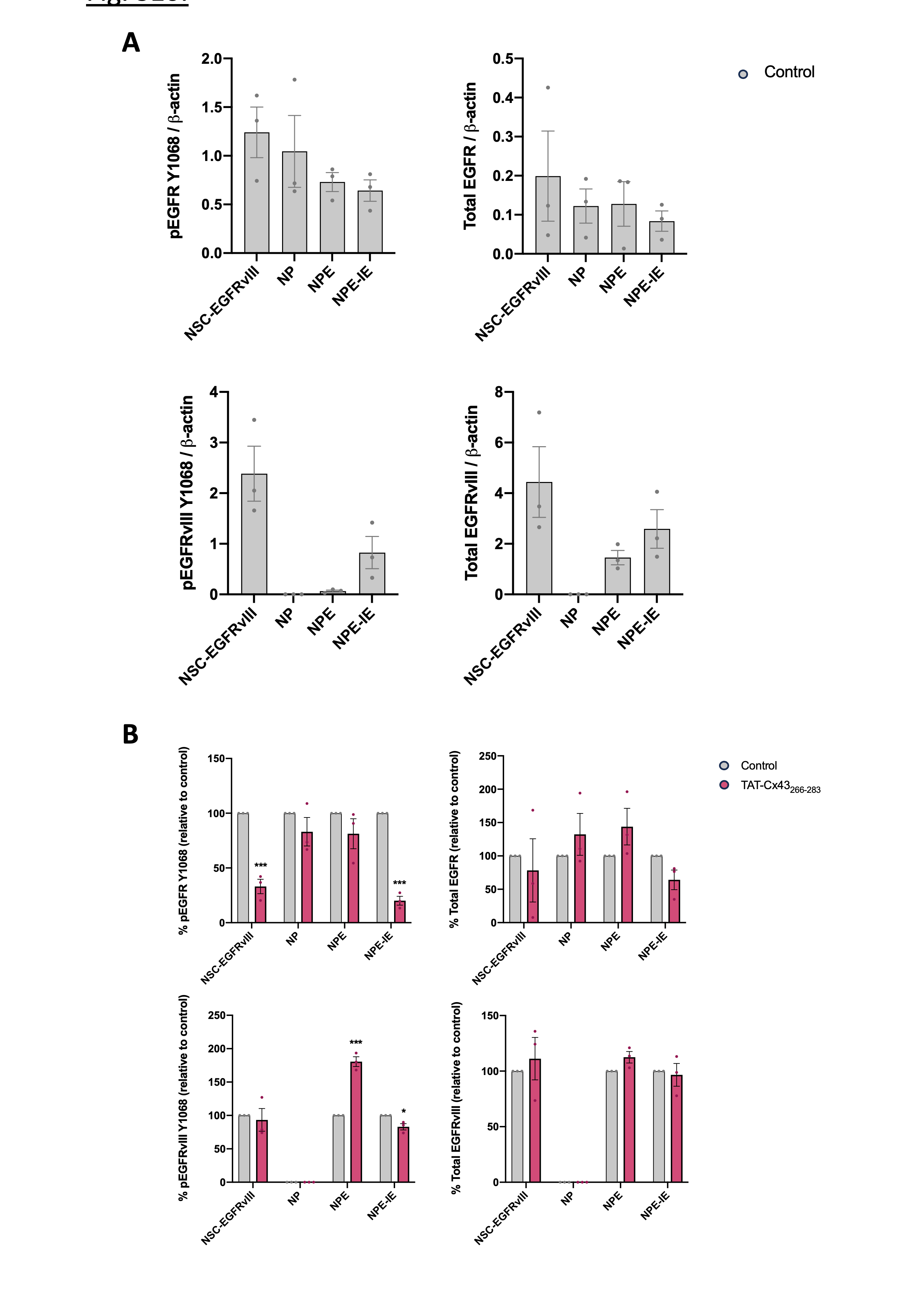

Supplement: noae060_suppl_Supplementary_Materials [file noae060_suppl_supplementary_materials.zip › Supplementary material/FigS10.tiff]

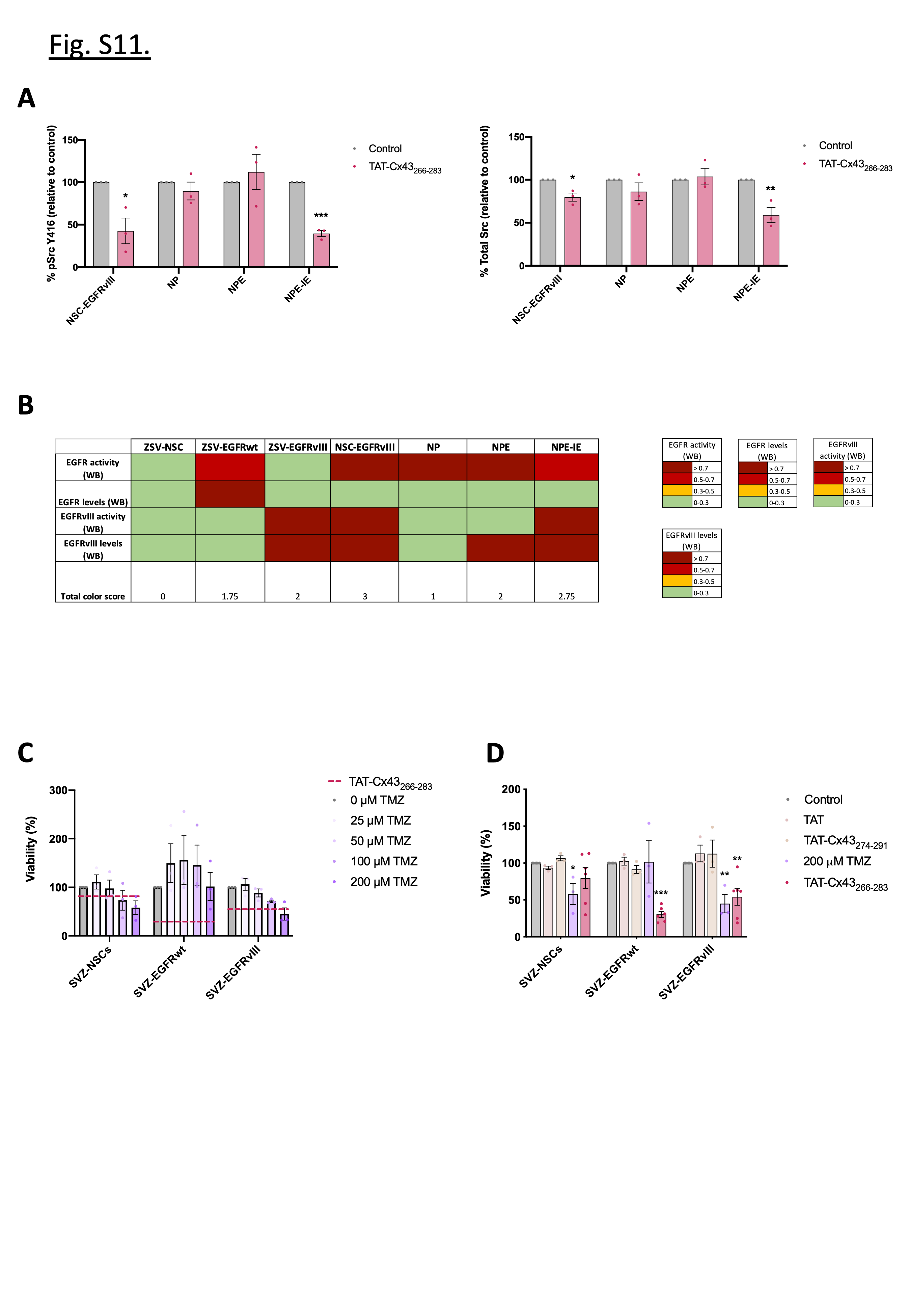

Supplement: noae060_suppl_Supplementary_Materials [file noae060_suppl_supplementary_materials.zip › Supplementary material/FigS11.tiff]

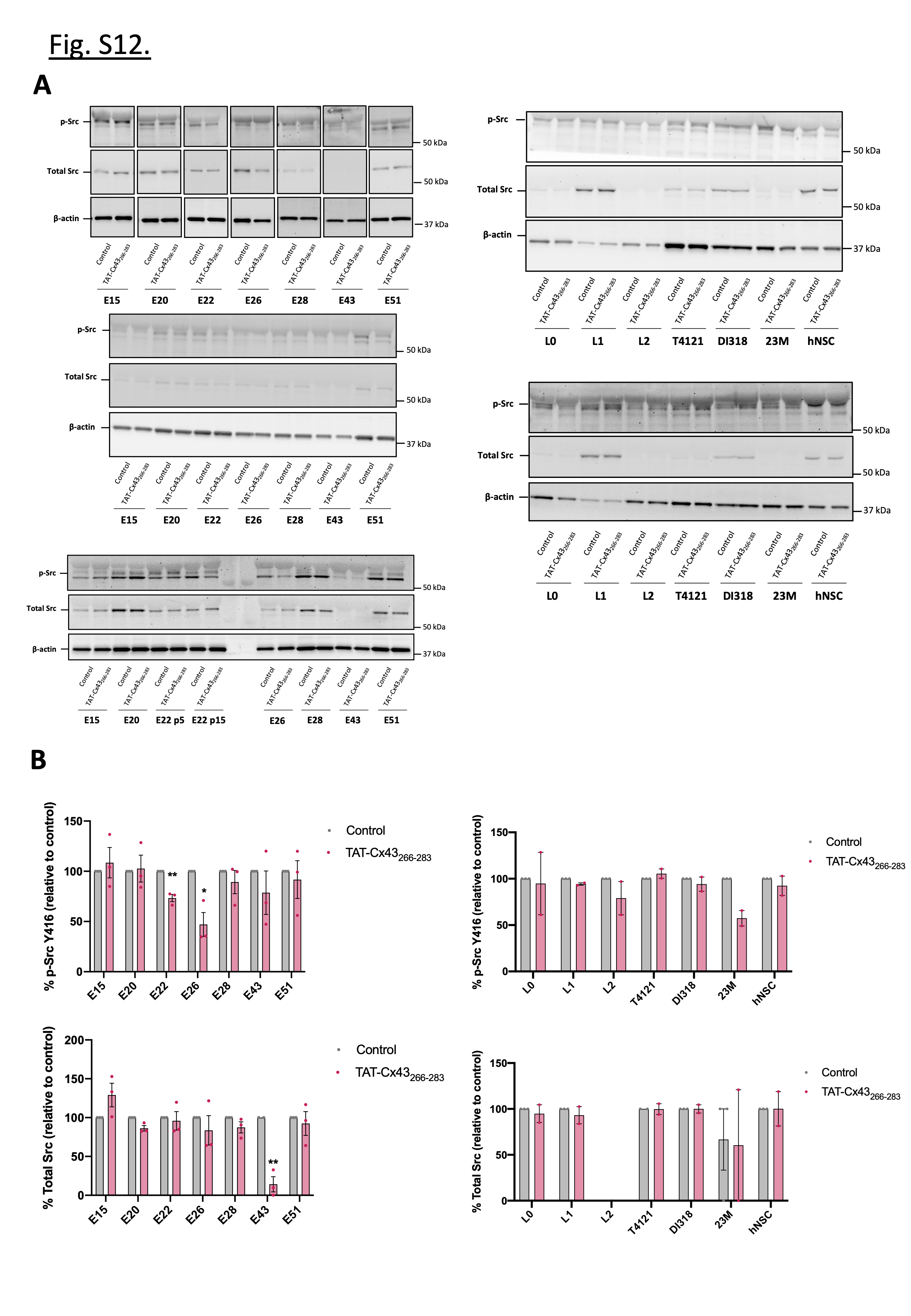

Supplement: noae060_suppl_Supplementary_Materials [file noae060_suppl_supplementary_materials.zip › Supplementary material/FigS12.tiff]

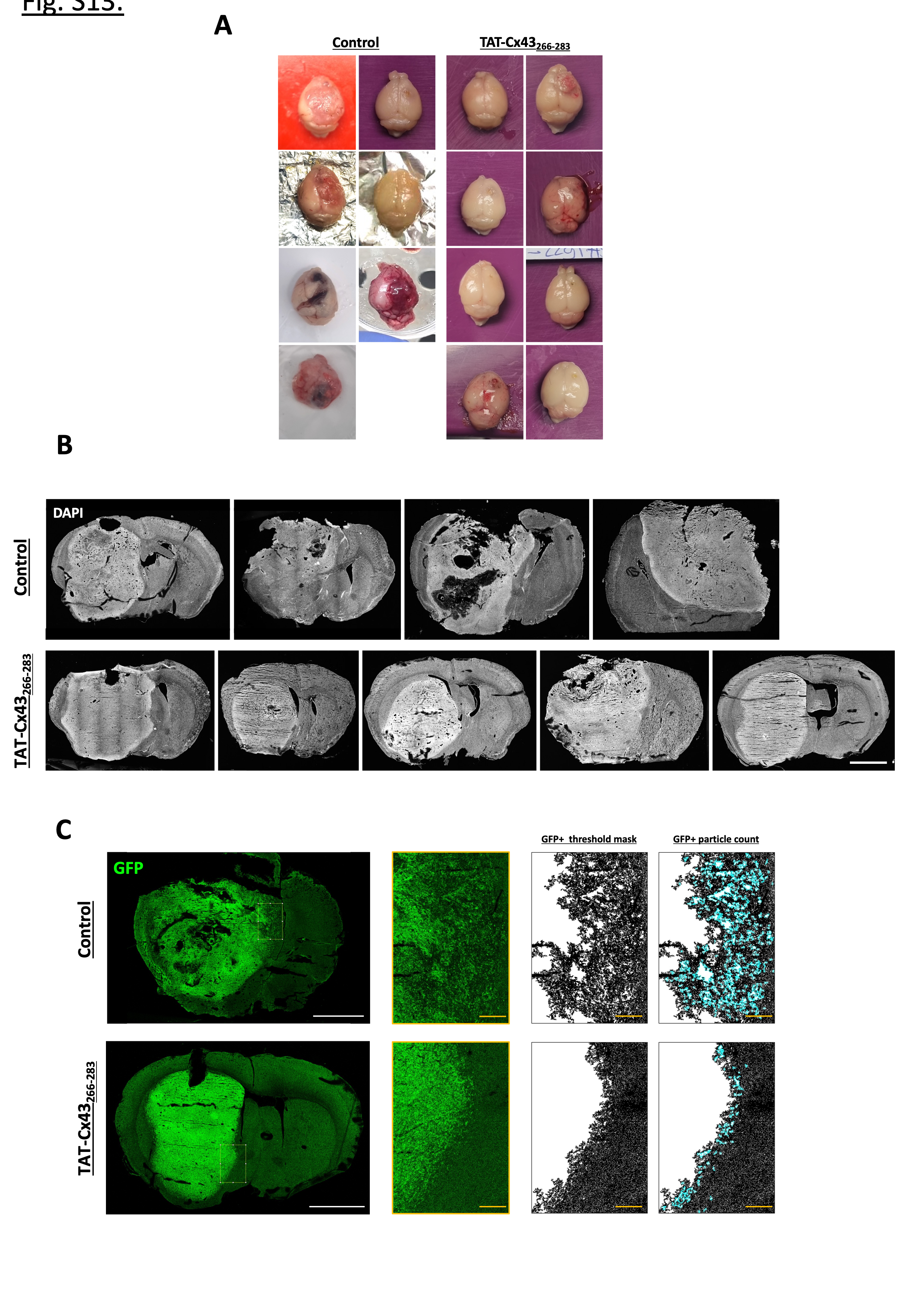

Supplement: noae060_suppl_Supplementary_Materials [file noae060_suppl_supplementary_materials.zip › Supplementary material/FigS13.tiff]

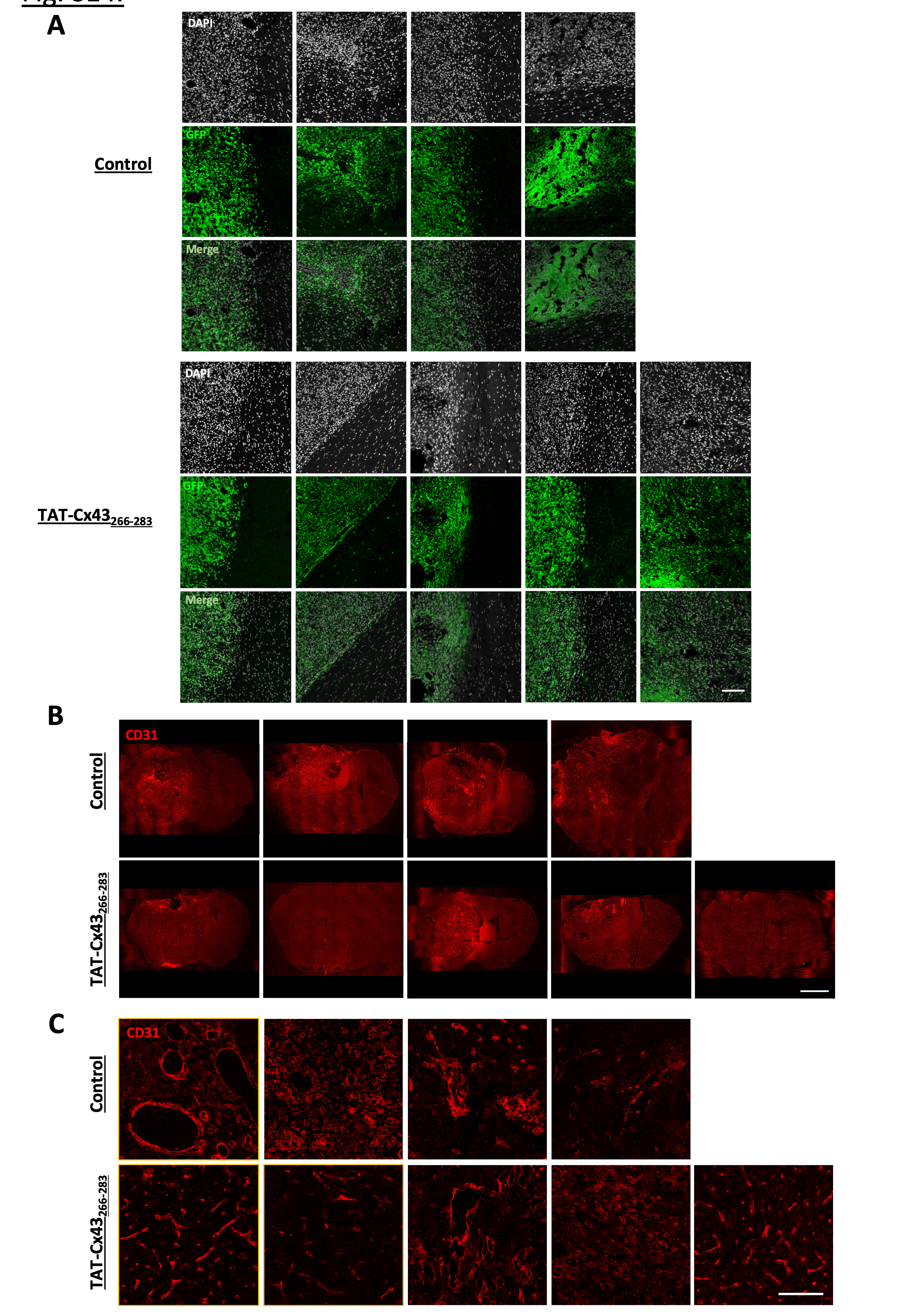

Supplement: noae060_suppl_Supplementary_Materials [file noae060_suppl_supplementary_materials.zip › Supplementary material/FigS14.tiff]

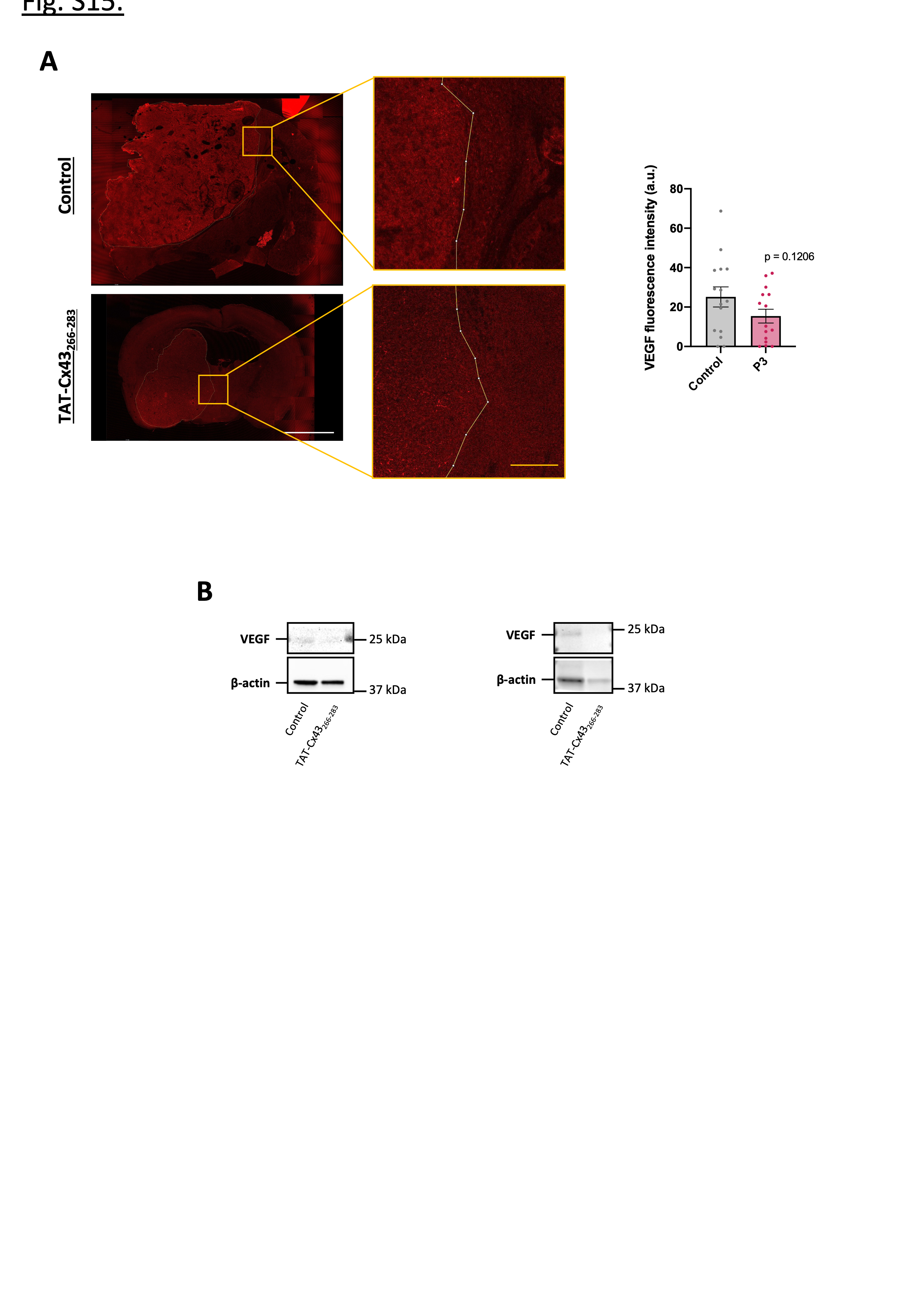

Supplement: noae060_suppl_Supplementary_Materials [file noae060_suppl_supplementary_materials.zip › Supplementary material/FigS15.tiff]

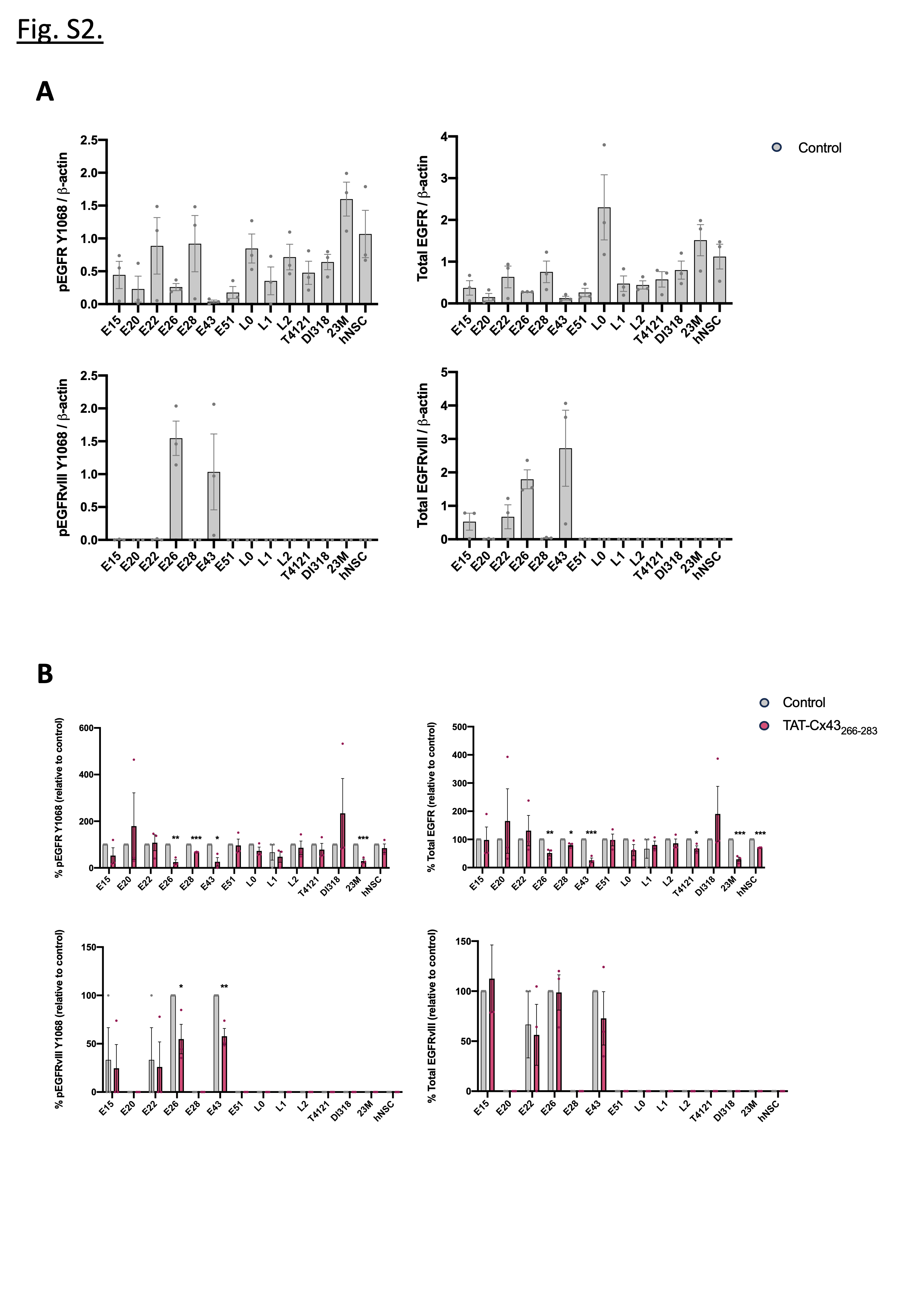

Supplement: noae060_suppl_Supplementary_Materials [file noae060_suppl_supplementary_materials.zip › Supplementary material/FigS2.tiff]

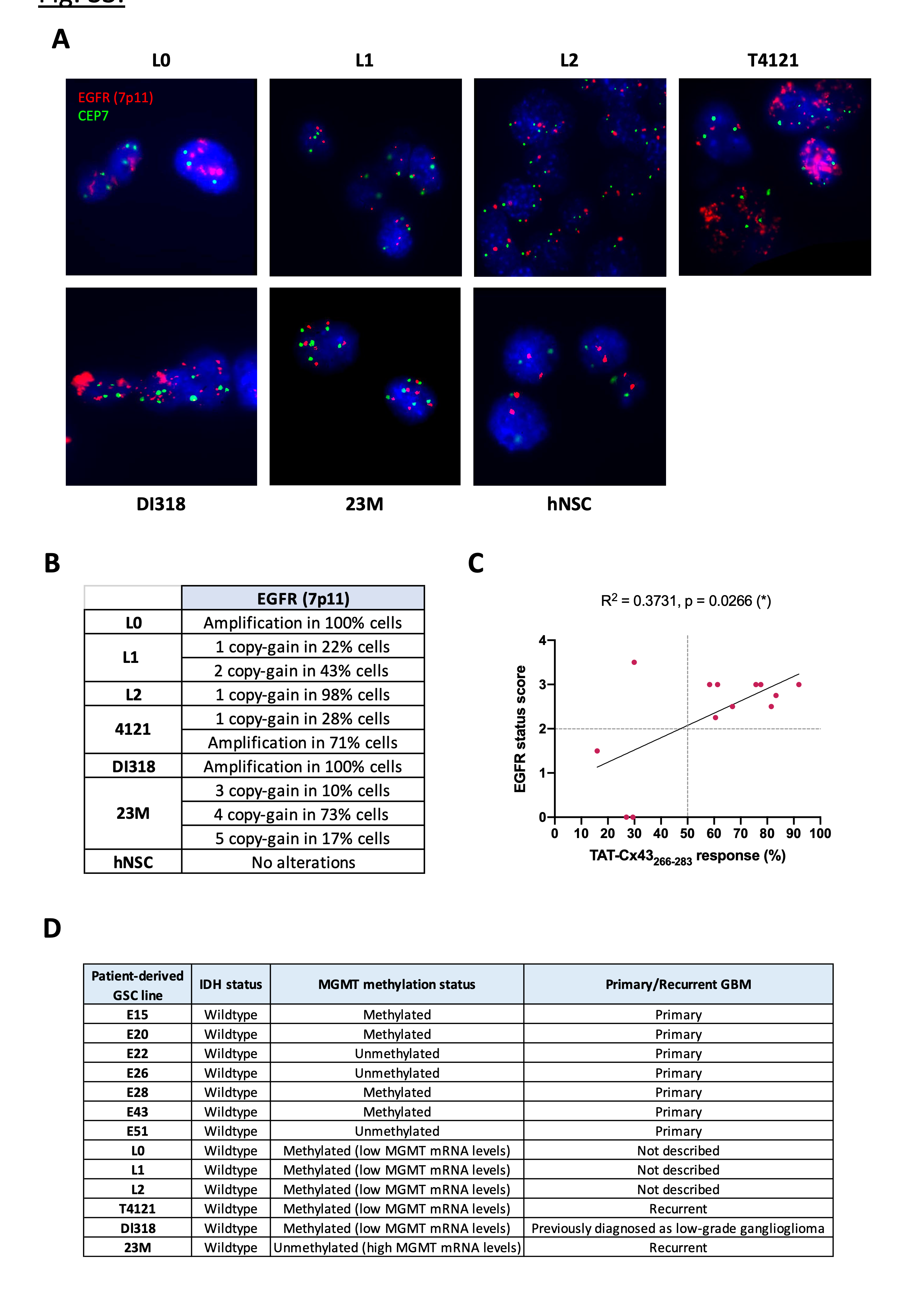

Supplement: noae060_suppl_Supplementary_Materials [file noae060_suppl_supplementary_materials.zip › Supplementary material/FigS3.tiff]

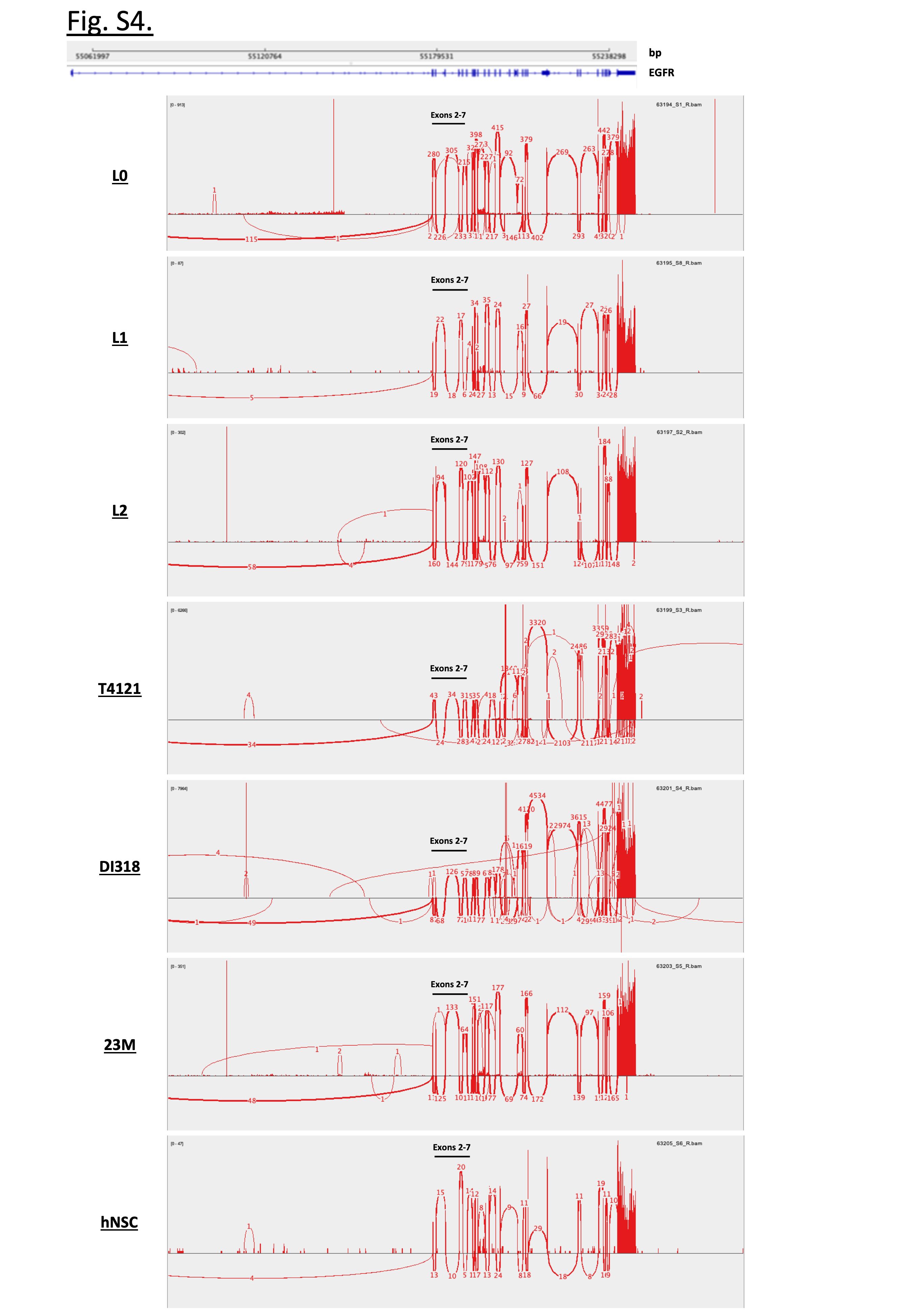

Supplement: noae060_suppl_Supplementary_Materials [file noae060_suppl_supplementary_materials.zip › Supplementary material/FigS4.tiff]

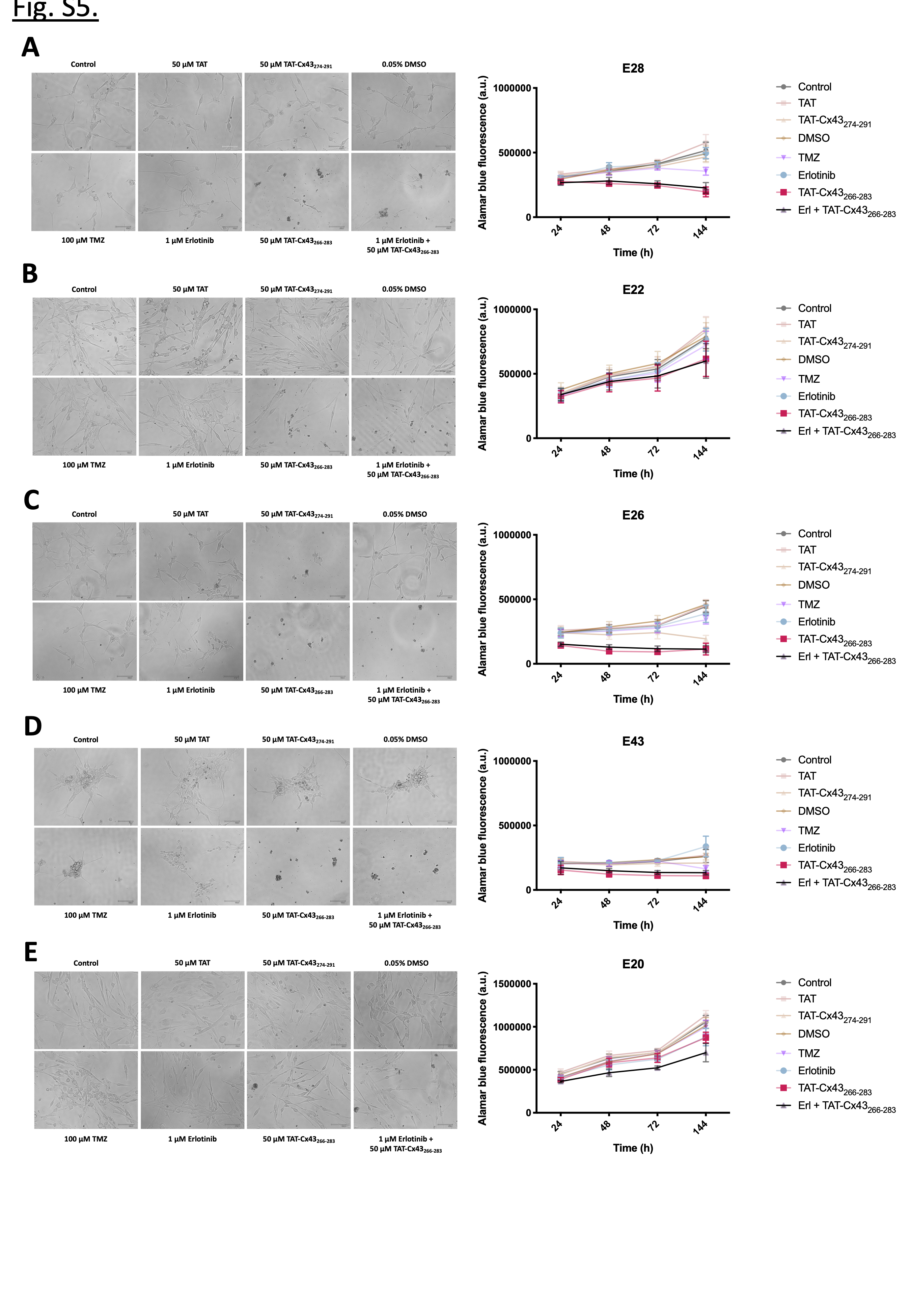

Supplement: noae060_suppl_Supplementary_Materials [file noae060_suppl_supplementary_materials.zip › Supplementary material/FigS5.tiff]

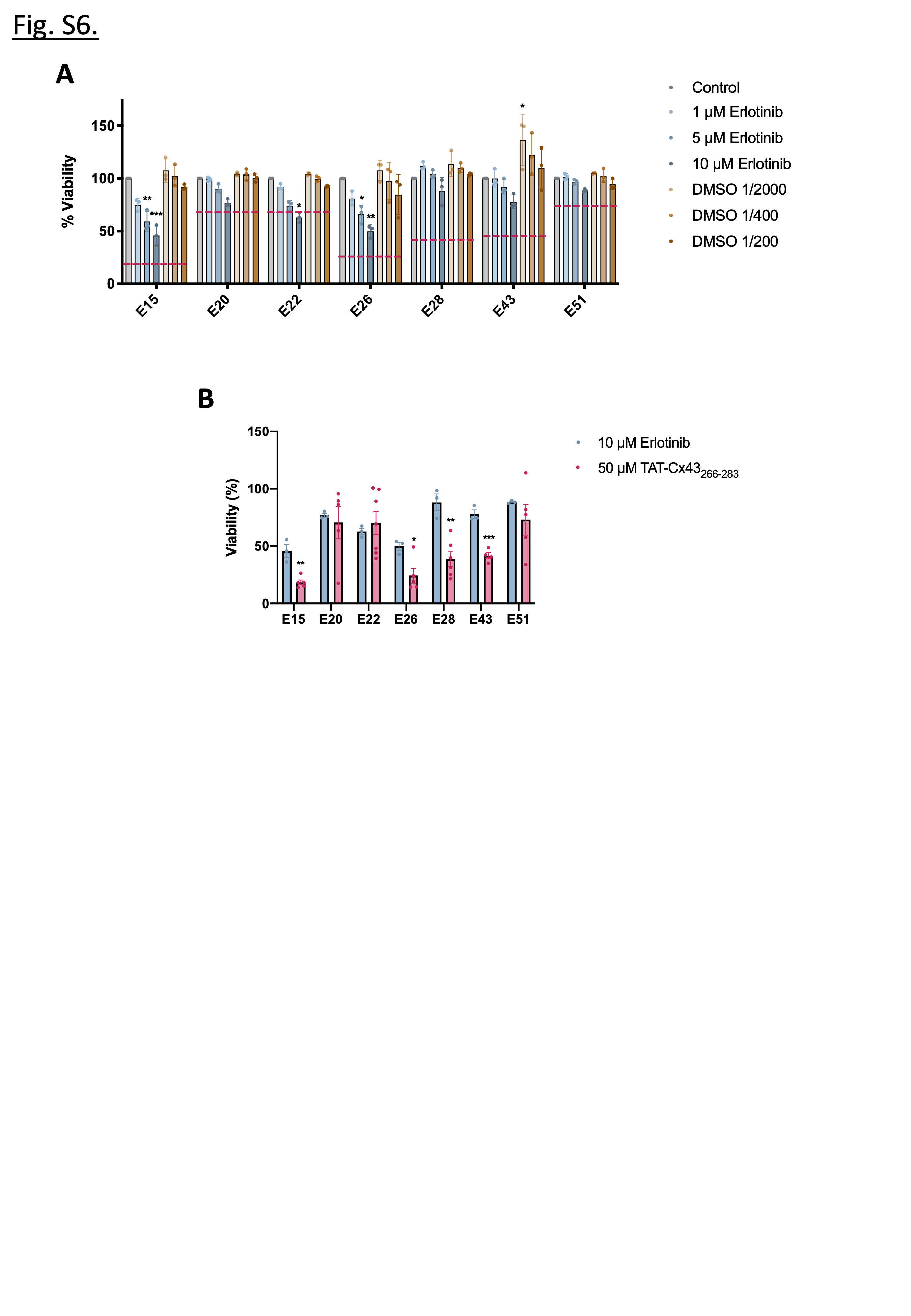

Supplement: noae060_suppl_Supplementary_Materials [file noae060_suppl_supplementary_materials.zip › Supplementary material/FigS6.tiff]

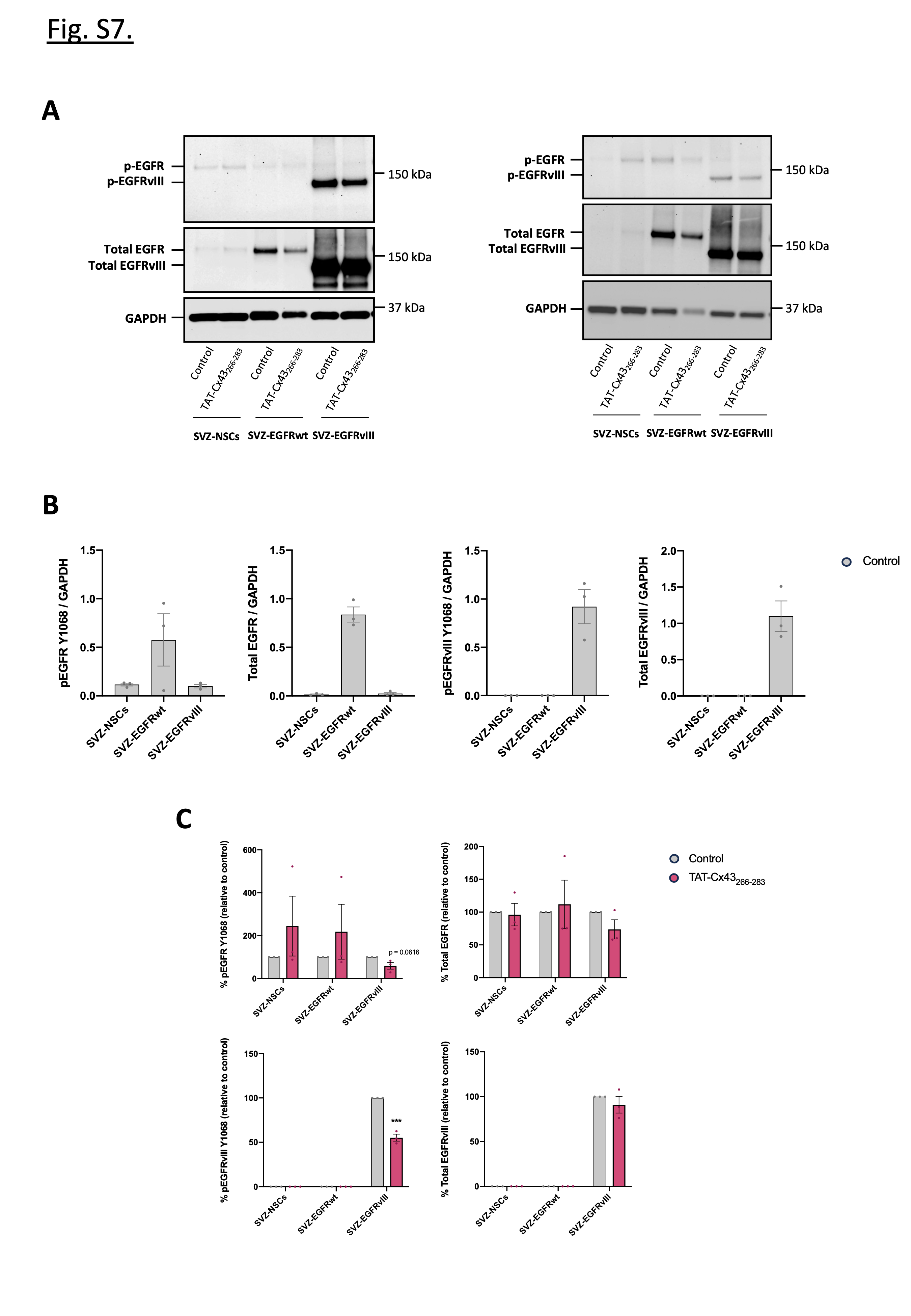

Supplement: noae060_suppl_Supplementary_Materials [file noae060_suppl_supplementary_materials.zip › Supplementary material/FigS7.tiff]

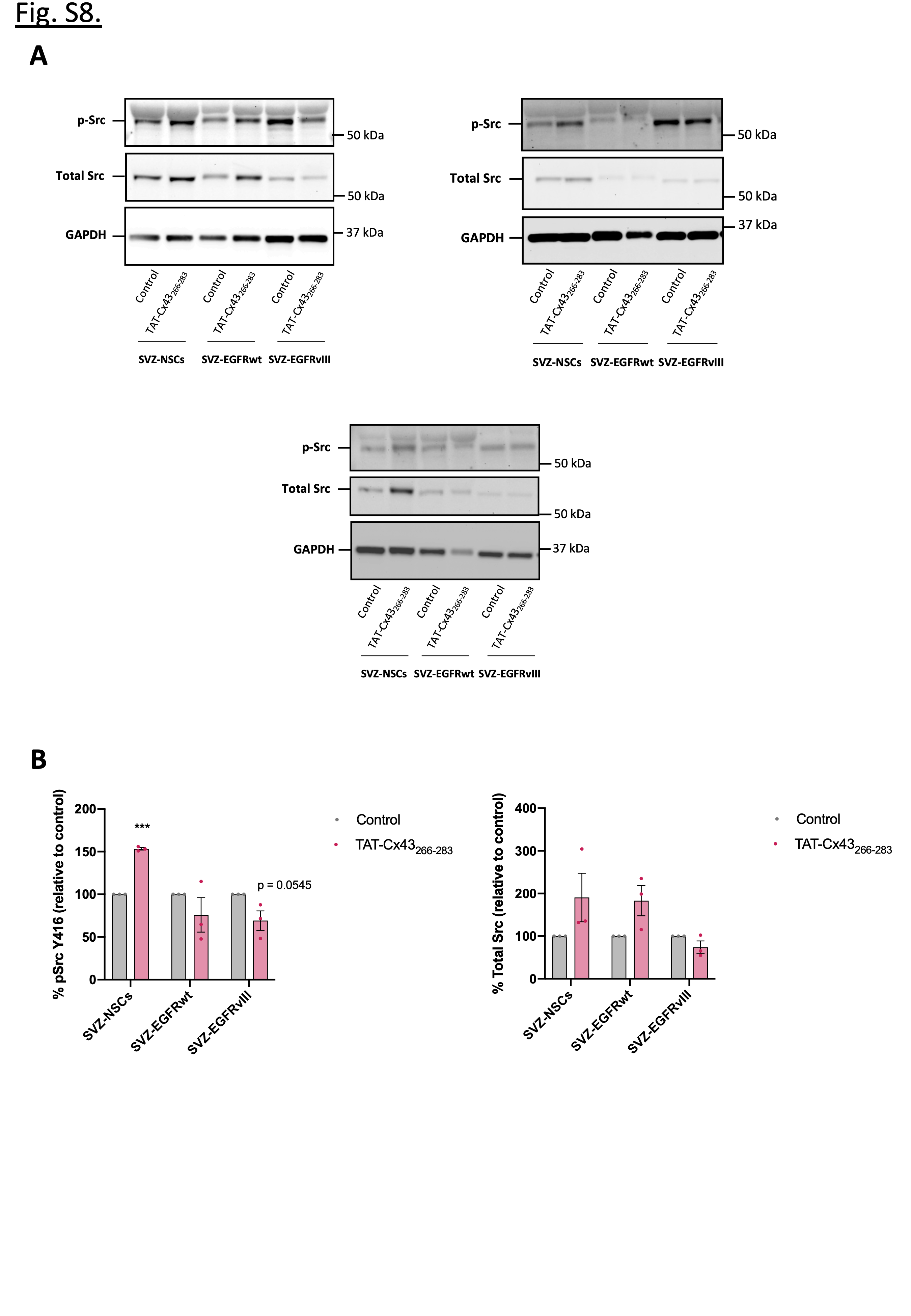

Supplement: noae060_suppl_Supplementary_Materials [file noae060_suppl_supplementary_materials.zip › Supplementary material/FigS8.tiff]

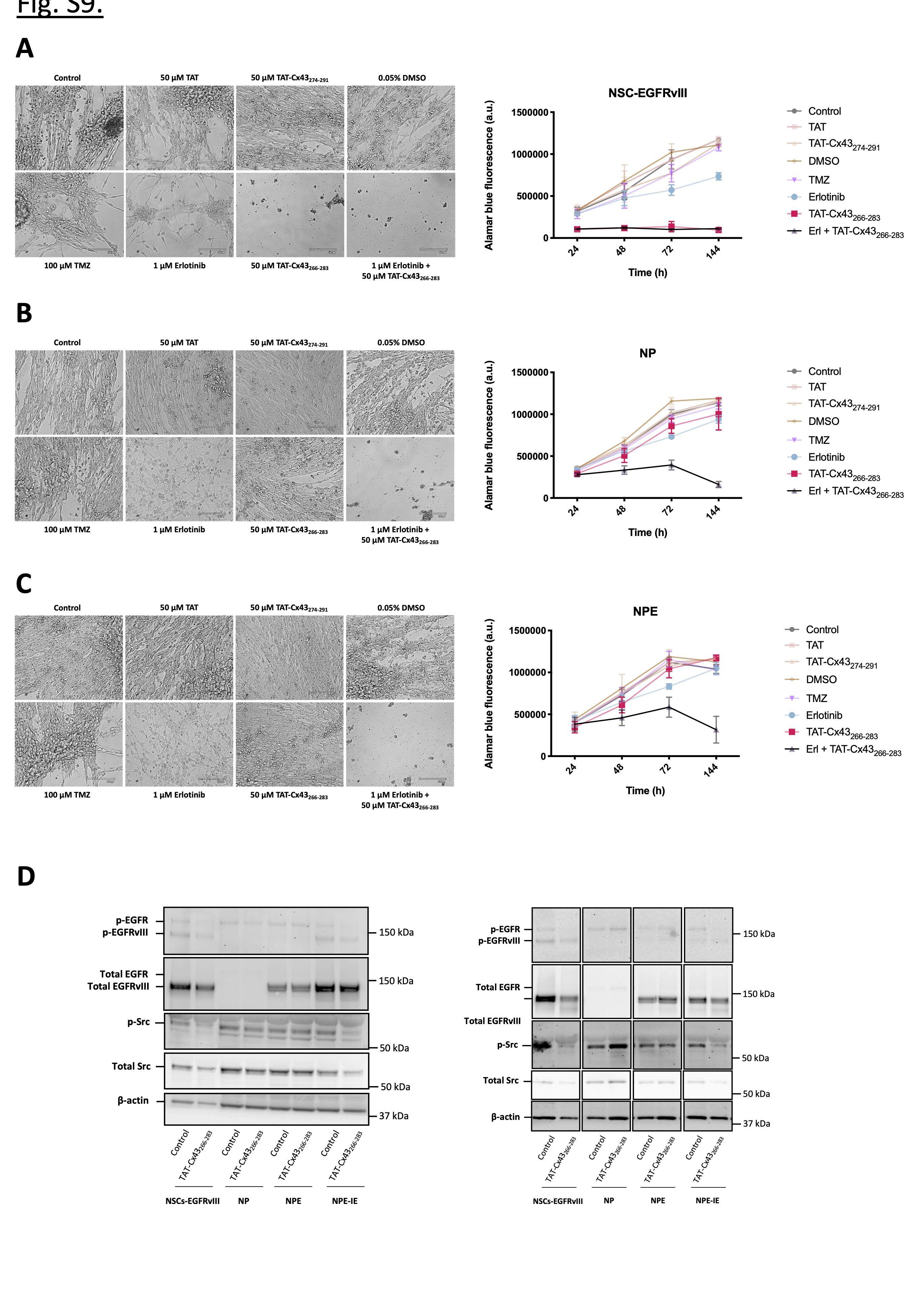

Supplement: noae060_suppl_Supplementary_Materials [file noae060_suppl_supplementary_materials.zip › Supplementary material/FigS9.tiff]
